# Single-Cell and Spatial Transcriptomics Reframe the Immunosuppressive Microenvironment of Neuroendocrine Neoplasms

**DOI:** 10.3390/cancers18132176

**Published:** 2026-07-07

**Authors:** Yoshihiro Takahashi, Shin Tsunekawa

**Affiliations:** 1 Department of Diabetes, Endocrinology and Metabolism and Department of Rheumatology and Clinical Immunology, Gifu University Graduate School of Medicine, 1-1 Yanagido, Gifu 501-1194, Japan; 2 Department of Clinical Genetics Center, Gifu University Hospital, 1-1 Yanagido, Gifu 501-1194, Japan; 3 Center for One Medicine Innovative Translational Research, Gifu University, 1-1 Yanagido, Gifu 501-1194, Japan

**Keywords:** neuroendocrine neoplasms, single-cell RNA sequencing, spatial transcriptomics, tumor microenvironment, immunosuppression, precision immuno-oncology

## Abstract

Neuroendocrine neoplasms (NENs) are a heterogeneous family of tumors that have traditionally been regarded as “immune cold” and largely refractory to PD-1/PD-L1 checkpoint blockade, with notable exceptions such as Merkel cell carcinoma (MCC). The advent of single-cell RNA sequencing (scRNA-seq) and spatial transcriptomics has enabled unprecedented dissection of the NEN tumor microenvironment (TME), but a cross-subtype synthesis is lacking. This review aims to integrate single-cell and spatial transcriptomic findings across major NEN subtypes to reframe NEN immunosuppression and delineate translational implications. To this end, we performed a structured narrative review of PubMed-indexed studies up to 30 April 2026, prioritizing original human scRNA-seq, single-nucleus RNA-seq, spatial transcriptomic, and spatial proteomic studies of NENs, supplemented by mechanistic, clinical, and biomarker-focused reports providing essential context. Across these studies, synthesis spanning pancreatic, pulmonary, gastrointestinal, cutaneous, pituitary, adrenal, and other NEN subtypes highlights conserved features beyond the PD-1/PD-L1 axis, including myeloid-dominated infiltration with alternative checkpoints (VISTA, TIM-3, Galectin-9), cancer-associated fibroblast-mediated immune exclusion, lineage-state-dependent immune visibility, and direct immunomodulation by neuroendocrine secretory products such as calcitonin gene-related peptide. We propose a four-layer framework integrating these mechanisms and linking them to emerging biomarkers and therapies, including DLL3-directed bispecifics, alternative checkpoint inhibitors, stromal-targeting agents, and peptide receptor radionuclide therapy combinations. Together, these findings indicate that single-cell and spatial transcriptomic studies reframe NEN immunosuppression as a multilayered, subtype-dependent process, providing a conceptual scaffold for biomarker-guided, subtype-adapted therapeutic strategies and prospective clinical trial design in neuroendocrine oncology.

## Simple Summary

Neuroendocrine neoplasms are a diverse group of tumors that can arise in many parts of the body, including the pancreas, lung, gut, skin, and pituitary gland. Although their incidence is rising, most patients with these tumors do not respond well to modern immune-based therapies, because the tumors are often considered “immune cold.” Recent technologies that examine individual tumor cells and their precise locations within tissues have begun to reveal why this is the case. In this review, we synthesize findings from these new approaches across all major subtypes of neuroendocrine neoplasms and propose a unifying four-layer framework that explains how these tumors evade immune attack. We highlight how this framework can guide the development of new biomarkers and therapies, including approaches beyond conventional checkpoint inhibitors. Our aim is to help researchers and clinicians design more rational, subtype-specific strategies to improve outcomes for patients with these challenging tumors.

## 1. Introduction

Neuroendocrine neoplasms (NENs) constitute a diverse family of neoplasms arising from the diffuse neuroendocrine system, with the capacity to develop in virtually any anatomical site [1,2]. They encompass well-differentiated neuroendocrine tumors (NETs) and poorly differentiated neuroendocrine carcinomas (NECs), a distinction that is central to contemporary classification and clinical management.

Over the past four decades, the incidence of NETs has risen dramatically, increasing approximately 6.4-fold from 1973 to 2012 in the United States, with age-adjusted rates reaching 6.98 per 100,000 persons [1]. This trend is observed across both low-grade and high-grade NENs and spans all primary sites, though more recent Surveillance, Epidemiology, and End Results (SEER)-based analyses of NETs across all sites suggest a plateau at approximately 9.39 per 100,000 since 2015 [3]. The rising incidence, coupled with significant geographic disparities—for example, China reports an incidence of only 1.14 per 100,000 compared with 6.26 per 100,000 in the United States [4]—underscores the growing global clinical burden of these tumors.

Despite treatment advances, survival outcomes vary markedly by stage and subtype: SEER-based analyses indicate substantially more favorable outcomes for localized well-differentiated NETs than for distant-stage or poorly differentiated gastroenteropancreatic NENs [1,5], underscoring the urgent need for improved treatment strategies and predictive biomarkers.

The 2022 World Health Organization (WHO) classification system has established a unified framework that distinguishes well-differentiated NETs (grades G1–G3) from poorly differentiated neuroendocrine carcinomas (NECs), incorporating Ki-67 proliferation indices and molecular markers across organ systems [6]. This taxonomy has been further refined for specific sites, including transcription factor-based classification of pituitary neuroendocrine tumors (PitNETs) [7], genetic susceptibility-driven classification of pheochromocytomas and paragangliomas (PPGLs) [8], and molecular reclassification of pulmonary neuroendocrine neoplasms [9,10].

The recognition that G3 NETs and NECs represent biologically distinct entities—with NECs showing alterations such as RB1 and PTEN mutations, while G3 NETs retain the mutational landscape of their lower-grade counterparts [11]—has had profound implications for treatment selection and immunotherapy eligibility.

Despite advances in classification, the therapeutic landscape for many NENs, particularly well-differentiated NETs, remains constrained by a fundamental biological characteristic: these tumors have long been considered “immune cold.” This designation reflects several convergent features, including a characteristically low tumor mutational burden (TMB)—with pancreatic NETs showing TMB-high rates as low as 1.3% [12]—limited CD8+ T cell infiltration compared with epithelial carcinomas [13], heterogeneous and generally low PD-L1 expression [14,15], HLA class I (HLA-I) downregulation in pancreatic NENs, particularly in higher-grade or poorly differentiated contexts where supported by available data [16], and pronounced tumor vascularity, which may contribute to immune exclusion or immune compartmentalization in some contexts, although this relationship remains incompletely established in NETs [14,17].

The clinical corollary of this immune cold phenotype has been the disappointing performance of PD-1/PD-L1 checkpoint inhibitors in unselected NET populations, with objective response rates (ORRs) as low as 3.7% in the KEYNOTE-158 trial [18].

However, this monolithic designation obscures substantial heterogeneity. Merkel cell carcinoma (MCC), a neuroendocrine skin cancer, has shown marked sensitivity to PD-1/PD-L1 blockade in pivotal prospective studies, with first-line pembrolizumab achieving an objective response rate of 56% [19] and first-line avelumab achieving an objective response rate of 62% [20]. In contrast, combination immunotherapy in high-grade neuroendocrine neoplasms has shown activity in selected settings, with a subgroup analysis of high-grade lesions in the nonpancreatic NET cohort of the DART trial reporting a 44% objective response rate [21] and a related high-grade neuroendocrine neoplasm cohort reporting an objective response rate of 26% [22].

These discrepancies indicate that the tumor microenvironment (TME) of NENs is far more heterogeneous than previously appreciated, and that conventional profiling methods—such as immunohistochemistry (IHC) and bulk RNA sequencing—have been insufficient to capture this complexity.

The advent of single-cell RNA sequencing (scRNA-seq) and spatial transcriptomics has revolutionized our ability to interrogate the TME. Since the landmark application of scRNA-seq to dissect multicellular tumor ecosystems [23], these technologies have been progressively applied to NEN subtypes, yielding transformative insights into cellular heterogeneity, intercellular communication, and spatial immune architecture. Several recent reviews have addressed individual facets of this landscape: Sen et al. provided a comprehensive overview of pulmonary neuroendocrine neoplasms, with particular focus on small cell lung cancer (SCLC) molecular biology and therapeutic strategies [10], Cives et al. reviewed the NET TME from a conventional perspective [14], and organ-specific analyses have synthesized findings for individual subtypes. However, a cross-subtype synthesis of single-cell and spatial transcriptomic data across neuroendocrine neoplasms remains limited.

This review aims to fill that gap by providing a comprehensive synthesis of scRNA-seq and spatial transcriptomics studies across all major NEN subtypes, to reframe the immunosuppressive TME beyond the PD-1/PD-L1 axis, and to delineate the translational implications for biomarker development, therapeutic targeting, and treatment selection in clinical practice.

## 2. Methodological Framework

### 2.1. Single-Cell RNA Sequencing: Principles and Platforms

Single-cell RNA sequencing (scRNA-seq) resolves individual cells within heterogeneous tissues, avoiding the averaging of bulk approaches [23]. In NEN research, the 10x Genomics Chromium system has predominated, applied across pancreatic NETs [24,25], SCLC [26,27], pituitary NETs [28,29], cervical NECs [30,31], and olfactory neuroblastoma [32,33]. These studies resolved the tumor, immune, and stromal compartments of the NEN microenvironment.

Single-nucleus RNA sequencing (snRNA-seq) has become an important complementary approach, particularly for archived or difficult-to-dissociate specimens such as pheochromocytomas and paragangliomas [34] and pancreatic NETs [35], while complementary scRNA-seq has also been applied to hormone-secreting pheochromocytoma [36].

### 2.2. Spatial Transcriptomics: Preserving Tissue Architecture

Spatial transcriptomics preserves tissue architecture, localizing gene expression within intact sections. In NEN research, the Visium platform (10x Genomics) has been the most widely used spatial approach and has been applied to prostate neuroendocrine carcinoma (NEPC) [37] and pituitary NETs [38].

Bulk transcriptomic profiling of insulinomas identified a low-endocrine subtype characterized by reduced YY1 expression and diminished insulin synthesis signatures; this finding was further supported by 10× Visium HD spatial transcriptomic analysis showing lower insulin scores in the low-endocrine subtype than in the endocrine subtype [39].

Other spatially resolved proteo-transcriptomic approaches have been used in SCLC [40] and gastric mixed adenoneuroendocrine carcinoma (MANEC) [41], while higher-resolution methods have been applied to MCC [42] and mixed neuroendocrine-non-neuroendocrine neoplasms (MiNEN) [43]. Spatial proteomic approaches have further complemented these analyses, particularly in studies of tertiary lymphoid structures in MCC [44].

Integrative studies in PitNETs [28,38] and SCLC [40,45] have shown how single-cell reference atlases can support spatial deconvolution and reconstruction of local cellular interactions, thereby linking transcriptional states to tissue organization.

### 2.3. Computational Approaches: Deconvolution, Cell–Cell Interaction, and Multi-Omics Integration

Deconvolution tools such as BayesPrism for bulk RNA-seq [46] and cell2location [47] and RCTD [48] for spatial data infer cell-type composition from complex tissues.

Cell–cell communication analyses, commonly performed using CellChat [49] or related ligand-receptor frameworks, have been central to identifying functionally relevant signaling axes in NENs, including macrophage-tumor interactions in PitNETs [28,29] and fibroblast-tumor crosstalk in pancreatic NETs [50].

Multi-omics integration—combining scRNA-seq with ATAC-seq, proteomics, or methylation—has refined neuroendocrine subtype classification and lineage-state modeling, particularly in neuroendocrine prostate cancer [51,52,53]. Pan-tissue DNA methylation atlases have further supported cell-type decomposition [54].

### 2.4. Review Scope and Source Selection

This narrative review synthesizes recent evidence on single-cell and spatial transcriptomic studies in neuroendocrine neoplasms, with emphasis on tumor microenvironmental features, biomarker discovery, and therapeutic implications. Relevant articles were identified primarily through PubMed searches up to 30 April 2026, using combinations of disease-related terms for major neuroendocrine neoplasm subtypes together with technology-related terms including “single-cell RNA sequencing”, “single-nucleus RNA sequencing”, “spatial transcriptomics”, “spatial proteomics”, and “multi-omics”, and microenvironment-related terms including “tumor microenvironment”, “immune microenvironment”, “immunosuppression”, “checkpoint”, “macrophage”, “dendritic cell”, “cancer-associated fibroblast”, and “tertiary lymphoid structure”.

Priority was given to original human studies using single-cell, single-nucleus, spatial transcriptomic, or spatial proteomic approaches. Conventional molecular studies, preclinical reports, clinical trials, and review articles were included selectively when they provided essential disease-specific context, mechanistic validation, or translational interpretation.

Conference abstracts without sufficient methodological detail and studies not directly relevant to neuroendocrine neoplasms, neuroendocrine differentiation, or tumor-immune-stromal interactions were generally excluded. Additional studies were identified through reference list screening. The aim was not to provide an exhaustive systematic review but rather a focused narrative synthesis of conceptually influential and clinically informative studies.

## 3. Single-Cell Atlases Across Neuroendocrine Neoplasm Subtypes

The following section summarizes single-cell and spatial transcriptomic atlases by anatomical subtype (Table 1) to preserve disease-specific context. Rather than isolated catalogs, these studies provide the empirical basis for the cross-organ immunosuppressive principles synthesized in Section 4, including lineage-state-dependent immune visibility, myeloid-centered suppression, stromal exclusion, and neuroendocrine secretory immunomodulation.

### 3.1. Pancreatic Neuroendocrine Tumors

#### 3.1.1. Tumor-Intrinsic Heterogeneity and Metastatic Programs

Pancreatic NETs (pNETs), well-differentiated NETs, have been among the most extensively profiled NET subtypes by single-cell approaches. The pioneering study by Zhou et al. applied scRNA-seq to primary and metastatic pNETs, showing spatiotemporal heterogeneity with progressive activation of hypoxia, metabolic reprogramming, and proliferative pathways during malignant progression [24]. A two-gene signature comprising PCSK1 and SMOC1 was identified as predictive of metastatic potential, suggesting potential translational utility as a biomarker [24].

This finding was extended by Yin et al., who characterized a metastasis-associated PCSK1+ neuroendocrine subpopulation regulated by the ATF6 transcription factor and exhibiting specific interactions with tumor fibroblasts [50].

Arbesfeld-Qiu et al. performed single-nucleus RNA-seq on primary and metastatic pNET specimens, discovering that macrophage-derived glutamate functions as a paracrine signal promoting both immunosuppression and tumor cell migration [35]. This finding highlights a metabolic axis of TME communication previously unappreciated in pNETs.

Ye et al. further reported that histological grade and genomic instability patterns are associated with distinct core activation pathways at the single-cell level, and identified hepatic metastasis-associated features involving tip cells, lymphatic endothelial cells, cancer-associated fibroblasts (CAFs), macrophages, CD1A+ dendritic cells, Tregs, MAIT cells, and ILCs [25].

In the functional subtype of insulinomas, transcriptomic profiling identified a novel “low-endocrine” subtype characterized by reduced YY1 expression, linking transcription factor activity to insulin production capacity [39].

#### 3.1.2. Immune and Stromal Microenvironment

An important immunological dimension was added by Young et al., who analyzed 207 pNET samples and identified four molecular subtypes (MLP-1, MLP-2, insulinoma-like, intermediate) with a 132-gene immune signature [55]. The MLP-1 subtype, comprising 26–31% of cases, exhibited an “immune-high” phenotype linked to a hypoxia-necroptosis cascade activating viral mimicry and STING pathways, resulting in T cell inflammatory gene expression and macrophage infiltration; the latter was confirmed by multiplex immunofluorescence [55]. This finding identifies a pNET subpopulation that may be amenable to immunotherapy, providing a molecular basis for patient selection beyond conventional grading.

Mechanistic studies have indicated that pNET cells secrete apolipoprotein E (ApoE) to induce tip endothelial cells, thereby promoting CAF recruitment and stromal remodeling [56]. In preclinical models, mTOR inhibition suppressed ApoE secretion, suggesting that stromal remodeling may represent one mechanism through which mTOR pathway inhibition influences pNET biology [56].

Additional studies have shown that small extracellular vesicle-derived miR-183-5p promotes SPP1-dependent macrophage M2 polarization through the PDCD4/PI3K-gamma/AKT/mTOR pathway [57], whereas hypoxic tumor-derived exosomal miR-4488 promotes liver metastasis through macrophage M2 polarization via RTN3/FABP5 suppression [58].

Complementing these microenvironment-focused findings, Ji et al. identified four proteomic subtypes with distinct immune landscapes and therapeutic vulnerabilities in non-functional pNETs, nominating CDK5 and CACNA1D as druggable targets, validated in organoid and xenograft models [59].

In the same context, proteomics-based prognostic modeling was proposed for non-functional pNETs [59], while separate NEN-focused studies have highlighted HLA-I dysregulation and mutation-based genomic features as additional candidate prognostic factors [16,60]. Enhancer profiling using H3K27ac ChIP-seq further delineated ARX- and PDX1-associated epigenomic subtypes with distinct clinical outcomes [61].

Collectively, these studies position pNETs as a prototypical model of multilayered immunosuppression in well-differentiated NENs, in which tumor-intrinsic endocrine programs, macrophage-centered metabolic communication, endothelial remodeling, and CAF recruitment may converge to shape metastatic potential and immune exclusion.

### 3.2. Pulmonary NENs: Small Cell Lung Cancer, LCNEC, and Pulmonary Carcinoids

SCLC, the prototypical poorly differentiated NEC, represents the most extensively studied high-grade neuroendocrine carcinoma at single-cell resolution. The landmark atlas by Chan et al. profiled 155,098 cells from 21 SCLC specimens, showing greater transcriptomic diversity than non-small cell lung cancer and identifying a PLCG2-high phenotype with stem-like properties and poor prognosis [26]. This atlas provided the framework for understanding intratumoral heterogeneity in SCLC.

The molecular subtyping of SCLC has been refined through single-cell and transcriptomic studies, with ASCL1, NEUROD1, and POU2F3 transcription factor-defined subtypes, and Gay et al. subsequently defined a fourth inflamed subtype (SCLC-I) rather than the previously proposed YAP1 subtype [62,63].

Ireland et al. indicated that MYC may drive temporal evolution between subtypes, promoting transition from ASCL1+ states toward NEUROD1+ or YAP1+ states [64]. Zhang et al. further showed that ASCL1+ and NEUROD1+ cells coexist within individual tumors, with NEUROD1+ cells exhibiting EPCAM negativity and enhanced metastatic capacity [65]. This phenotypic plasticity may have important therapeutic implications, as subtype switching could alter drug sensitivity and immune visibility.

Spatial transcriptomics has provided critical context for understanding SCLC immune heterogeneity. Jin et al. combined single-cell and spatial proteo-transcriptomic profiling of 44 SCLC tumors, identifying the REST transcription factor as a biomarker of reduced neuroendocrine features associated with enhanced antitumor immunity and improved survival [40].

Tian et al. showed that non-neuroendocrine SCLC cells exhibit increased inflammatory gene signatures and immune cell infiltration, features that may be associated with greater immunotherapy sensitivity [66]. Desai et al. reported that cancer-associated fibroblasts contribute to immune exclusion and poor prognosis, with the TME composition associated with neuroendocrine cell plasticity and therapeutic resistance [45].

In pulmonary carcinoids (well-differentiated pulmonary NETs), Bischoff et al. characterized a non-inflammatory, monocyte-derived myeloid cell population as the dominant immune component, with stromal and immune cells showing paracrine interactions through NOTCH, VEGF, TGF-beta, and JAK/STAT signaling [69].

In large cell neuroendocrine carcinoma (LCNEC), a poorly differentiated NEC, Stewart et al. reported that YAP1 status stratifies tumors into mesenchymal/inflammatory (YAP1-high) and SCLC-like/immune-cold (YAP1-low) subtypes, suggesting differential sensitivity to immunotherapy versus SCLC-directed strategies [70]. Ji et al. used spatial transcriptomics to identify SMC1A overexpression in combined LCNEC and functionally validated its role in promoting migration, invasion, and G1/S phase transition while suppressing apoptosis in lung cancer cell-line models [71]. Complementary digital immunophenotyping of pulmonary atypical carcinoids and LCNEC identified three immune-defined clusters with prognostic significance, with PD-L1 tumor expression strongly associated with the immune-high, poor-prognosis LCNEC-enriched cluster [72].

Together, pulmonary NEN studies illustrate how neuroendocrine lineage plasticity and stromal organization jointly influence immune visibility, providing a conceptual bridge between transcription factor-defined tumor states and spatially organized immune exclusion.

### 3.3. Gastrointestinal NETs

Gastrointestinal NETs (predominantly well-differentiated NETs, in contrast to the poorly differentiated gastrointestinal NECs discussed below) have been the subject of several recent single-cell investigations. Deng et al. established a comprehensive single-cell atlas of colorectal NETs with liver metastases, identifying stress-like immune phenotypes and COLEC11+ matrix CAFs enriched in metastatic sites and predictive of adverse prognosis [73]. This study highlighted receptor-ligand interactions as candidate therapeutic pathways in the metastatic microenvironment.

For small intestinal NETs (SI-NETs), Somech et al. profiled 10 tumors by scRNA-seq, identifying epithelial-like and neural-like subtypes [74]. Notably, neuroendocrine cells were largely non-proliferative, whereas higher proliferation was observed mainly in B and plasma cells, particularly in the epithelial-like subtype, challenging assumptions about the proliferative dynamics of well-differentiated NETs [74].

Axling et al. reported transcriptional reprogramming in SI-NET cells, with loss of epithelial differentiation regulators and acquisition of neural signaling pathways [75]. Sei et al. identified a specific enterochromaffin cell subset within crypts as the cell of origin for SI-NETs, with PRODH2 serving as a biomarker and UCHL1/MBD3L2 upregulated during tumorigenesis [76].

Site-specific immune patterns have been documented by Cives et al., who demonstrated that duodenal NETs display adaptive immune resistance mechanisms distinct from jejunal and ileal tumors, underscoring the importance of anatomical context in interpreting TME data [15].

In the gastric setting, gene expression and immune-profiling analysis of mixed adenoneuroendocrine carcinoma (MANEC) showed distinct molecular and microenvironmental profiles between adenocarcinoma and NEC components, with NEC regions showing increased fibroblast presence and proliferative pathway activation [41]. Zhou et al. showed that neuroendocrine transdifferentiation in gastric cancers (a transdifferentiated NEC) is accompanied by progressive downregulation of interferon pathways, suggesting a mechanism that may contribute to immune evasion during NE differentiation [77].

Chen et al. showed that colorectal NEC exhibits a predominantly “cold” immune profile, though CCL5 expression correlated with improved CD8+ infiltration and survival [78]. Although appendiceal NETs currently lack single-cell data, conventional pathological studies have described tumor-infiltrating lymphocytes with possible prognostic relevance, supporting this subtype as a priority for future single-cell investigation [79].

Thus, gastrointestinal NENs highlight the importance of anatomical context, metastatic niche formation, and component-specific immune architecture, suggesting that immune-cold phenotypes in this group may arise through both lineage-dependent and site-specific mechanisms.

### 3.4. Merkel Cell Carcinoma as a Cutaneous Neuroendocrine Carcinoma

MCC, a cutaneous and frequently virus-associated neuroendocrine carcinoma, occupies a unique position among NENs as one of the most immunotherapy-responsive subtypes. Reinstein et al. performed a multi-modal analysis of 116 patients with MCC, comprising 186 samples, discovering that preexisting tissue-resident memory CD8+ and V-delta-1 gamma-delta T cells predict immunotherapy response [80]. Responders showed type I/II interferon upregulation and clonal T cell expansion, while non-responders showed increased tumor proliferation and neural marker expression [80].

Lei et al. applied high-resolution spatial transcriptomics to uncover epidermal-dermal divergences in MCC, showing that epidermotropic MCC cells adopt keratinocyte differentiation programs through p63 activity, consistent with microenvironment-dependent phenotypic adaptation [42].

The role of myeloid cells in MCC has been characterized by Tabachnick-Cherny et al., who showed that immunosuppressive myeloid populations, including CD163+, CD14+, and S100A8+ TAM subsets, were enriched in tumors with substantial CD8+ T-cell infiltration, while no preferential spatial interaction between TAMs and CD8+ T cells was observed when comparing tumors with different treatment responses [81].

Ohnezeit et al. identified a novel immune evasion mechanism in virus-positive MCC, where the Merkel cell polyomavirus small T antigen suppresses type I interferon signaling through IRF9 downregulation [82]. Spatial proteomic studies in MCC have linked tertiary lymphoid structure (TLS) density and organized B-cell/T-cell neighborhoods with immunotherapy response, although the precise cellular features reported differ across studies [44,83].

In contrast to most well-differentiated NENs, MCC illustrates that neuroendocrine lineage does not inevitably imply immune ignorance; rather, viral antigenicity, tissue-resident lymphocyte circuits, and TLS formation can remodel the NEN TME toward an immune-responsive state.

### 3.5. Pituitary Neuroendocrine Tumors

PitNETs (well-differentiated NETs) have been the subject of particularly comprehensive single-cell characterization. The landmark study by Su et al. integrated scRNA-seq of 177,000 cells with spatial transcriptomics across 57 specimens, identifying aggressive tumor clusters and suggesting that SPP1+ tumor-associated macrophages may promote invasion through an inferred SPP1-ITGAV/ITGB1 axis [28]. This finding identifies a specific macrophage-tumor interaction as a candidate therapeutic axis in aggressive PitNETs.

Lin et al. characterized three tumor immune microenvironment (TIME) subtypes across PitNET lineages, with CX3CR1+ macrophages inferred to regulate tumor cell apoptosis through an INHBA-ACVR1B axis [29]. Yan et al. identified heterogeneous TAM clusters with distinct functions and associated PBK overexpression with increased tumor proliferation and migration [84].

Wang et al. combined spatial and single-cell approaches to somatotroph PitNETs, showing DLK1/RCN1 overexpression in copy-number-high regions and TGF-beta pathway involvement in progression [38]. Martinez-Mendoza et al. applied spatial transcriptomics to dopamine agonist-resistant lactotroph tumors, identifying PI3K/AKT pathway activation and lipid metabolism alterations associated with treatment resistance [85].

Zhang et al. developed scRNA-seq-based differentiation state markers with potential utility for predicting PitNET recurrence, with poorly differentiated tumors showing higher recurrence rates in most lineages, although the pattern may differ in the SF1 lineage [86].

### 3.6. Pheochromocytoma and Paraganglioma

Pheochromocytomas and paragangliomas (PPGLs)—neural-crest-derived neuroendocrine tumors that fall outside the epithelial NET/NEC dichotomy—exhibit distinctive molecular subtypes with implications for immune profiling. Zhang et al. conducted pioneering scRNA-seq of an ectopic adrenocorticotropic hormone (ACTH)/corticotropin-releasing hormone (CRH)-secreting pheochromocytoma, identifying a novel chromaffin-like cell type simultaneously producing ACTH and CRH [36].

Brazda et al. reported extensive patient-to-patient transcriptome heterogeneity using snRNA-seq, with RET and SDHB mutations driving distinct molecular programs reflecting different developmental arrest stages [34].

Calsina et al. characterized the genomic and immune landscape of metastatic PPGLs, showing a broadly immunosuppressive microenvironment linked to genomic subtypes, with subtype-specific exceptions, and nominating immune parameters as prognostic and predictive biomarkers [87].

Qin et al. identified two molecular subtypes—metabolic (NDUFA4L2/COX4I2 markers, immunosuppressive) and kinase (RET/PNMT markers, HLA-I deficient)—with distinct therapeutic implications: the kinase subtype shows HLA-I downregulation potentially regulated by RET signaling, supporting the hypothesis that kinase inhibitor–immunotherapy combinations may be worth exploring, whereas the metabolic subtype may require different therapeutic approaches because of retained antigen-presenting capacity despite an immunosuppressive milieu [88]. Liu et al. extended this classification to three transcriptional subtypes with distinct immune landscapes: subtype C1 was immunosuppressive with poor prognosis, while subtype C2 showed CD8+ T cell infiltration, a feature that may be compatible with greater immunotherapy sensitivity [89].

Berends et al. showed that metastatic pheochromocytomas exhibit TNF-alpha/TGF-beta signaling activation detectable in primary tumors, suggesting potential utility for earlier metastatic risk stratification [90]. Wang et al. constructed a 302,696-cell atlas of adrenal incidentalomas, including pheochromocytoma among other adrenal lesions, and reported myeloid cell dominance in benign lesions versus lymphocyte predominance in malignant tumors [91].

Overall, PPGLs provide a clear example of subtype-dependent immune architecture, in which kinase-driven antigen-presentation defects and metabolic-subtype immunosuppression may require distinct therapeutic strategies rather than a uniform immunotherapy approach.

### 3.7. Other Neuroendocrine Neoplasm Entities

Olfactory neuroblastoma (ONB). Yang et al. performed scRNA-seq on 10 ONB tumors, identifying five expression programs and proposing a neural/basal/mesenchymal three-category classification, with immunosuppressive macrophages nominated as therapeutic targets [32].

Notably, Finlay et al. showed that ONB mimics the molecular heterogeneity and lineage trajectories of SCLC, sharing NEUROD1+ and POU2F3+ states and phenotypic plasticity [33]. This finding suggests that lineage programs related to the SCLC transcriptional framework, particularly NEUROD1- and POU2F3-associated states, may be conserved across anatomically disparate NENs.

Zunitch et al. identified the globose basal cell of the olfactory epithelium as the cell of origin, with NEUROD1 as a differential marker and EZH2 as a therapeutic target [92]. Xue et al. demonstrated high SSTR2 expression in neural-type ONB, suggesting potential for peptide receptor radionuclide therapy (PRRT) [93].

Medullary thyroid carcinoma (MTC). Hou et al. provided the first comprehensive scRNA-seq-based TME atlas of MTC, comparing it with papillary thyroid carcinoma (PTC) [94]. Their analysis indicated that CGRP expressed by MTC tumor cells may drive dendritic cell dysfunction through cAMP-related pathway activation and KLF2 upregulation, leading to impaired T cell priming [94]. CGRP receptor antagonists restored DC development in vitro, identifying CGRP signaling as both a mechanism of neuroendocrine-specific immunosuppression and a potential therapeutic target [94].

This study suggests that MTC may serve as a useful model for understanding how neuroendocrine secretory products shape the immunosuppressive TME.

Cervical neuroendocrine carcinoma. Wang et al. conducted the first scRNA-seq study of small cell neuroendocrine cervical carcinoma (a poorly differentiated NEC), identifying tissue-specific transcription factor networks distinct from lung and gastrointestinal NECs [30]. Complementary single-cell studies have described ASCL1-, NEUROD1-, POU2F3-, and YAP1-related molecular heterogeneity in small cell neuroendocrine cervical carcinoma, with subtype-associated immune features and YAP1-related variables proposed as components of prognostic modeling [30,31].

Chen et al. traced the cellular origin to squamous epithelial cells, with consistent SOX2 amplification and human papillomavirus (HPV) infection [95]. He et al. identified elevated mTOR/ERBB4/NRG1 signaling and MIF/TGF-beta-associated immunosuppression as candidate therapeutic pathways [96].

Masuda et al. suggested through organoid studies that mixed tumors are clonally derived, with drug treatment inducing NEC-to-adenocarcinoma differentiation shifts [97].

Prostate neuroendocrine carcinoma. Neuroendocrine transdifferentiation of prostate cancer—the prototypical transdifferentiated NEC—represents a clinically significant resistance mechanism to androgen deprivation therapy. Rodarte et al. identified ASCL1 as essential for neuroendocrine differentiation in an RB1/TP53-deficient, MYC-overexpressing mouse model, showing that ASCL1 deletion abolished NE identity without affecting tumor growth, thereby supporting ASCL1 as a lineage-specific therapeutic vulnerability [98]. Romero et al. showed that ASCL1+ cells emerge from KRT8+ luminal cells and progress to heterogeneous neuroendocrine prostate cancer, with ASCL1 loss producing transient regression requiring timing-dependent intervention [99].

Chen et al. identified bifurcated ASCL1/ASCL2 mutually exclusive lineages through multi-omics analysis, with cellular reprogramming preceding neural program activation [51]. Han et al. showed that FOXA2 may drive lineage plasticity and KIT pathway activation, with KIT inhibition suppressing NEPC tumor growth [52].

Watanabe et al. applied Visium spatial transcriptomics to de novo NEPC, visualizing fibrosis-associated and neuroendocrine-region gene expression patterns within the NE microenvironment [37].

Neuroendocrine bladder cancer. Zang et al. used a multimodal cohort of primary neuroendocrine bladder cancers (poorly differentiated NECs) to show that, despite a considerable mutational burden, these tumors were typically immunologically inactive and most often displayed immune-excluded or immune-desert phenotypes. They further found that a subset of mixed tumors with concurrent urothelial histology exhibited an immune-infiltrated phenotype with prognostic relevance, suggesting that histologic composition may influence the local immune microenvironment [100].

Mixed neuroendocrine-non-neuroendocrine neoplasms (MiNEN). Weiss et al. applied spatial transcriptomics to MiNEN, showing that morphological compartments align with transcriptomic profiles [43]. Their findings suggest that NEC components may exhibit relatively reduced immune-related pathway activity and elevated proliferative signaling, supporting the compartment-based WHO classification framework [43].

Although these subtype-specific atlases have substantially advanced the field, they should be interpreted with several caveats. Most datasets remain limited by small sample size, uneven representation of primary versus metastatic lesions, and differences in tissue dissociation, single-cell versus single-nucleus workflows, and spatial resolution. Moreover, many inferred ligand-receptor interactions and subtype-specific therapeutic vulnerabilities are based on computational prediction or limited experimental validation. Therefore, the atlases summarized above should be regarded as hypothesis-generating frameworks that require harmonized multi-institutional validation before being translated into routine clinical stratification.

## 4. Reframing the Immunosuppressive NEN TME

### 4.1. Immune Cell Landscape of NENs: Beyond PD-1/PD-L1

#### 4.1.1. Myeloid Compartment (TAMs, MDSCs, Dendritic Cells)

Single-cell studies have identified tumor-associated macrophages as a major immune population in the NEN TME and have shown functional heterogeneity invisible to conventional profiling.

In pituitary NETs (well-differentiated NETs), two distinct macrophage-tumor interaction axes have been characterized: SPP1+ macrophages inferred to promote invasion via the SPP1-ITGAV/ITGB1 axis [28], and CX3CR1+ macrophages inferred to regulate tumor cell apoptosis via the INHBA-ACVR1B axis [29]. The SPP1+ macrophage program may represent a recurrent feature across multiple NEN subtypes, as sEV-miR-183-5p from high-risk pancreatic NETs has been shown to induce SPP1-dependent M2 macrophage polarization through the PDCD4/PI3K-gamma/AKT/mTOR pathway [57].

In pancreatic NETs, macrophage-derived glutamate has been identified as a novel paracrine signal promoting both immunosuppression and tumor cell migration [35], indicating a metabolic communication axis not captured by conventional immune profiling. The previously described MLP-1 immune-high subtype suggests that macrophage infiltration in a subset of pNETs may arise through viral mimicry and STING pathway activation rather than neoantigen recognition [55].

In MCC (a cutaneous NEC), immunosuppressive TAM subsets are enriched in tumors with substantial CD8+ T-cell infiltration, suggesting that resistance to PD-1 pathway blockade may reflect an unfavorable myeloid-to-T-cell balance rather than a simple absence of immune engagement; however, preferential spatial co-localization between TAMs and CD8+ T cells was not demonstrated [81].

Dendritic cell dysfunction has, to date, been characterized as a specific immunosuppressive mechanism principally in medullary thyroid carcinoma (MTC) rather than as a generalizable pan-NEN feature. In medullary thyroid cancer, Hou et al. showed through scRNA-seq that CGRP impairs dendritic cell development through cAMP pathway activation and KLF2 upregulation, with CGRP receptor antagonists restoring immune function in vitro [94]. This mechanism is particularly relevant because it links the neuroendocrine secretory activity of tumor cells directly to TME immunosuppression and is supported by single-cell data.

It represents one of only a few subtype-specific examples—alongside glutamate signaling in pNET [35] and VGF–CAF coupling in SCLC [68]—that currently underpin Layer 4 as a candidate rather than an established, cross-NEN principle.

In pulmonary carcinoids (well-differentiated NETs), non-inflammatory monocyte-derived myeloid cells constitute the predominant immune infiltrate, engaging in paracrine interactions through NOTCH, VEGF, TGF-beta, and JAK/STAT pathways [69]. This non-inflammatory myeloid phenotype differs from the inflammatory macrophage populations seen in epithelial carcinomas and may reflect a distinct myeloid state in pulmonary carcinoids.

In a broader single-cell atlas of adrenal incidentalomas that included pheochromocytoma among other adrenal lesions, myeloid cell dominance was observed in benign lesions, whereas malignant tumors showed relative lymphocyte predominance [91]. These findings may suggest that myeloid-lymphocyte balance could have relevance in the broader adrenal tumor context.

#### 4.1.2. T Cell States: Exhaustion, Regulatory, and Alternative Checkpoints

Perhaps the most consequential single-cell discovery for NEN immunotherapy has been the identification of alternative immune checkpoint expression patterns. Hoffman et al. performed scRNA-seq of gastroenteropancreatic NETs and showed that infiltrating myeloid cells express VSIR (VISTA), HAVCR2 (TIM-3), LGALS9 (Galectin-9), and SIGLEC10, rather than PD-1/PD-L1 [101]. This finding offers a plausible molecular explanation for the limited efficacy of PD-1/PD-L1 blockade in well-differentiated GEP-NETs and highlights alternative checkpoint pathways as candidate therapeutic targets.

T cell exhaustion in the NEN TME exhibits subtype-specific features. In metastatic SCLC (a poorly differentiated NEC), malignant cells show reduced immune presentation function, and the tumor immune microenvironment exhibits immunosuppressive features [27]. The non-neuroendocrine SCLC subtype, characterized by YAP1-associated programs, shows increased inflammatory gene signatures and enhanced immune cell infiltration [66,70], consistent with the concept that neuroendocrine differentiation status inversely correlates with immune visibility.

In MCC, preexisting tissue-resident memory CD8+ and V-delta-1 gamma-delta T cells predict immunotherapy response, while non-responders show evidence of T cell failure with increased tumor neural marker expression [80].

In PPGLs, Qin et al. demonstrated that the kinase subtype exhibits HLA-I downregulation potentially mediated by RET signaling, while the metabolic subtype retains antigen-presenting capacity but creates an immunosuppressive milieu through alternative mechanisms [88], indicating subtype-specific T cell engagement patterns within a single NEN entity.

CAFs further modulate T cell function in the NEN TME. Lu et al. showed that the crosstalk between CAFs and non-neuroendocrine SCLC cells promotes glycolysis and the formation of antigen-presenting CAFs, which may paradoxically trap CD8+ T cells in stromal regions and contribute to regulatory T-cell recruitment, although this mechanism remains to be further validated [67]. This finding suggests a mechanism by which the stromal compartment may contribute to tumor tolerance through altered T-cell positioning and recruitment.

The clinical significance of T cell checkpoint expression is illustrated by the observation that Treg low density and CD8+TIM-3+ low density predict response to 177Lu-DOTATATE PRRT, integrating immune microenvironment parameters into theranostic decision-making [102].

#### 4.1.3. NK Cells, Gamma-Delta T Cells, and Innate Immunity

NK cell biology in the NEN TME remains less characterized than the adaptive immune compartment, though emerging data suggest a compromised innate immune landscape. Zhang et al. reported altered NK-cell states and an immunosuppressive immune microenvironment in metastatic SCLC through scRNA-seq, although the specific involvement of NK-cell exhaustion requires further validation [27]. The downregulation of interferon pathways reported during neuroendocrine transdifferentiation in gastric carcinoma [77] may, by extension, further impair NK-cell activation, although this has not been directly demonstrated in NK cells.

In MCC, the tissue-resident innate immune population characterized by Reinstein et al.—including V-delta-1 gamma-delta T cells—represents a distinct innate immune component that predicts immunotherapy response [80]. Gamma-delta T cells bridge innate and adaptive immunity and may be relevant in virus-positive MCC, although their precise functional contribution in this setting remains to be clarified. However, the role of gamma-delta T cells in non-MCC NEN subtypes has yet to be explored, representing a significant gap in our understanding of innate immunity in NENs.

The viral immune evasion mechanism identified in MCC—suppression of type I interferon signaling via IRF9 downregulation by the Merkel cell polyomavirus small T antigen [82]—further compromises innate immune surveillance. Whether analogous innate immune suppression mechanisms operate in virus-negative NENs remains an open question, though the CGRP-mediated DC dysfunction in MTC [94] raises the possibility that neuroendocrine secretory products may impair innate immunity in other NEN subtypes through distinct but potentially convergent mechanisms.

### 4.2. Cancer-Associated Fibroblasts in NENs: Heterogeneity and Functional Roles

#### 4.2.1. CAF Heterogeneity and Subtypes

Single-cell and spatial transcriptomics have described CAFs as a functionally diverse population with pleiotropic roles in the NEN TME. Desai et al. indicated that CAFs are principal contributors to immune exclusion in SCLC, with CAF abundance correlating with poor prognosis and non-neuroendocrine cell state reprogramming [45]. In colorectal NET liver metastases, COLEC11+ matrix CAFs were enriched in metastatic sites and predictive of adverse outcomes [73].

In non-NET solid tumor evidence, Forsthuber et al. characterized CAF heterogeneity in cutaneous cancers, including basal cell carcinoma, squamous cell carcinoma, and melanoma, describing myofibroblast-like RGS5+ CAFs, matrix CAFs, and immunomodulatory CAFs with functionally distinct roles in shaping the TME [103]; whether this taxonomy fully applies to NENs remains to be established.

The functional specialization of these subtypes suggests that CAF-targeting strategies may need to be tailored to the CAF composition of each NEN subtype and disease stage, rather than relying on a one-size-fits-all approach.

Verginadis et al. showed that the integrated stress response in stromal cells activates perivascular CAFs, driving collagen synthesis and amino acid metabolism that support angiogenesis and tumor progression [104]. This stress-responsive CAF activation may be particularly relevant in NENs, where hypoxic and highly vascular microenvironments may promote CAF-mediated immune exclusion.

#### 4.2.2. Metabolic Crosstalk and Translational Targeting

In SCLC, the metabolic crosstalk between CAFs and tumor cells has been elucidated at multiple levels. Ding et al. identified VGF as a regulator of ASCL1-driven neuroendocrine differentiation and showed that VGF promotes CAF activation and lactate exchange-mediated metabolic coupling between SCLC cells and CAFs, with combined targeting of VGF and MCT1 disrupting this interaction in preclinical models [68].

As noted in Section 4.1, Lu et al. suggested that antigen-presenting CAFs arising from CAF-tumor crosstalk may sequester CD8+ T cells and recruit regulatory T cells [67]. These findings collectively support an important role for CAFs in shaping the immunosuppressive NEN TME through metabolic, immune, and structural mechanisms.

In GI-NETs, gene expression and immune-profiling analysis of gastric MANEC showed that NEC components harbor increased fibroblast density compared with adenocarcinoma regions [41], while colorectal NET metastases show enrichment for matrix CAF populations [73]. These observations suggest that CAF enrichment may be a recurring feature of the NEN microenvironment in several gastrointestinal settings.

The translational relevance of CAF heterogeneity is supported by preclinical evidence that mTOR inhibition can suppress ApoE-mediated stromal remodeling in pNETs [56]. The identification of subtype-specific CAF vulnerabilities—COLEC11+ in metastatic GI-NETs, antigen-presenting CAFs in SCLC, and stress-responsive perivascular CAFs more broadly—may inform future stromal-targeting strategies.

### 4.3. Vascular and Endothelial Components of the NEN TME

Many well-differentiated NETs are characteristically highly vascularized tumors, and single-cell approaches have shown the complexity of the vascular compartment beyond what bulk analyses could discern. VEGF, VEGFR, FGF, and PDGF/PDGFR pathways drive NET angiogenesis and form the basis for approved anti-angiogenic therapies including sunitinib and surufatinib [17]. Single-cell studies have extended this understanding by identifying specific endothelial cell subtypes and their interactions with tumor cells.

Lou et al. showed in preclinical models that pNET-derived ApoE induces tip endothelial cells, which in turn recruit CAFs and remodel the tumor-stroma ratio [56]. Ye et al. suggested that endothelial components, particularly tip cells and lymphatic endothelial cells, are associated with the hepatic metastatic process in pNETs [25].

In prostate NEPC, spatial transcriptomics showed co-localization of fibrosis markers and vascular remodeling gene expression in neuroendocrine regions, supporting coordinated stromal-vascular remodeling in this phenotype [37]. In PPGLs, anti-angiogenic strategies including cabozantinib and other tyrosine kinase inhibitors have shown clinical activity, with combination approaches involving tyrosine kinase inhibitors (TKIs) plus immunotherapy or radionuclide therapy under investigation [105,106].

The highly vascular nature of PPGLs, together with emerging subtype-specific therapeutic hypotheses such as kinase inhibitor-immunotherapy combinations in kinase-type tumors [88], suggests that vascular biology may intersect with immune modulation in neuroendocrine tumors.

The vascular compartment also contributes to immune exclusion. The concept of CRATER (Cancer Regions of Antigen presentation and T cell Engagement and Retention) niches, defined as tumor cell surface niches or border zones at the tumor-immune interface, has been shown to predict immunotherapy response in melanoma and may provide a conceptual framework for other solid tumors [107], suggesting that the spatial relationship between vasculature, stroma, tumor cells, and immune cells may influence immune access and treatment responsiveness. Whether CRATER-like structures exist in NENs and how the characteristically dense vasculature of many well-differentiated NETs influences their formation remains to be determined.

### 4.4. Spatial Organization of the NEN TME: Immune Exclusion Patterns and Cell–Cell Interactions

Spatial transcriptomics has fundamentally advanced our understanding of how immune cells are organized within the NEN TME, moving beyond simple infiltration quantitation to topographic contextualization. Several spatial paradigms have been identified.

Immune exclusion. In SCLC, CAFs create physical barriers to immune cell infiltration, with spatial transcriptomics showing that CAF-rich regions correspond to immune-depleted tumor areas [45]. In MCC, epidermotropic tumor cells adopt keratinocyte-like programs within the epidermal microenvironment, consistent with microenvironment-dependent phenotypic adaptation that may influence immune recognition [42].

Immune compartmentalization. In a single case report, primary and metastatic carcinoid tumors showed heterogeneous immune microenvironments, with lower CD8+ infiltration in the metastatic lesion than in the primary lesion [108]. Colorectal NET liver metastases are enriched in stress-phenotype immune cells, suggesting that the metastatic microenvironment imposes specific immune cell states [73]. In pNETs, the MLP-1 “immune-high” subtype shows distinct spatial patterns of macrophage accumulation compared with other molecular subtypes [55], indicating that molecular classification has spatial correlates.

Tertiary lymphoid structures. TLSs have emerged as important spatial features associated with immunotherapy response, particularly in MCC. In MCC, TLS density correlates with treatment efficacy, with functional TLS containing high endothelial venules (HEVs) that recruit naive and central memory T cells [83]. Responders show dense T cell infiltration in close proximity to B cells within TLS [44], recapitulating findings in melanoma and other solid tumors in which CD8+/CD20+ co-localization [109] and switched memory B-cell enrichment [110] have been associated with immune checkpoint inhibitor (ICI) response. Organized TLS may help explain the relatively high immunotherapy responsiveness of MCC by supporting effective antigen presentation and T cell priming [44,83].

Cell–cell interaction networks. CellChat and ligand-receptor analyses have mapped specific interaction axes in NENs: the SPP1-ITGAV/ITGB1 axis in aggressive PitNETs [28], the INHBA-ACVR1B axis in PitNET macrophage-tumor communication [29], the PCSK1+ subpopulation-fibroblast interaction in metastatic pNETs [50], the MIF/TGF-beta immunosuppressive signaling in cervical NEC [96], and the CGRP-cAMP axis in MTC dendritic cell dysfunction [94]. These spatially resolved interaction networks identify candidate nodes for therapeutic investigation and illustrate how single-cell and spatial approaches can reveal vulnerabilities that are difficult to detect with bulk profiling.

Importantly, most of these axes were inferred from ligand-receptor co-expression and spatial proximity rather than established by functional perturbation; apart from the CGRP-cAMP axis in MTC, which was supported by receptor antagonism in vitro [94], they should be regarded as computationally nominated candidates that require experimental validation—a gap discussed further as a methodological limitation in Section 6.3 and taken up as a research priority in Section 7.

### 4.5. Cross-Organ Immunosuppressive Principles in NENs

**Figure 1:**
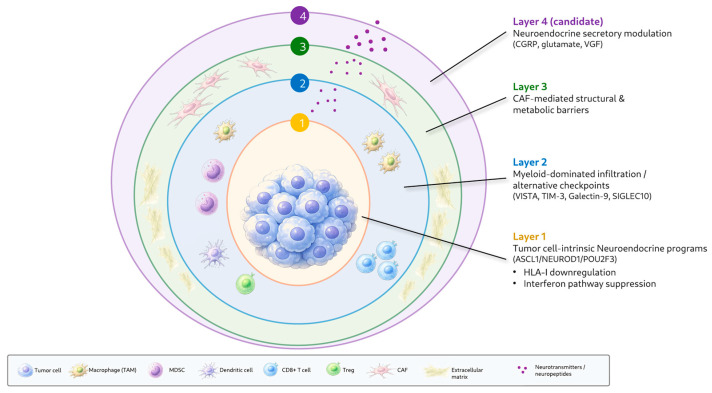
**Four-layer framework of immunosuppression in neuroendocrine neoplasms.** Single-cell and spatial transcriptomic studies indicate that the NEN tumor microenvironment is shaped by four interconnected layers of immunosuppression that vary across organs, histologic grades, and molecular subtypes. Layer 1 (Tumor cell-intrinsic neuroendocrine programs): lineage-defining transcription factors (ASCL1, NEUROD1, POU2F3) associated with HLA-I downregulation and impaired interferon signaling. Layer 2 (Myeloid-dominated immune infiltration with alternative checkpoint expression): TAM/MDSC enrichment, with alternative checkpoint molecules (VISTA, TIM-3, Galectin-9, SIGLEC10) predominating over the PD-1/PD-L1 axis. Layer 3 (CAF-mediated structural and metabolic barriers): subtype-specific CAF populations forming physical and metabolic barriers that exclude effector T cells. Layer 4 (Neuroendocrine secretory modulation): a candidate, NEN-distinctive principle of direct immunomodulation by secretory products (CGRP, glutamate, VGF), currently supported by a limited number of subtype-specific examples.

Despite their anatomical and clinical diversity, NENs share several conserved immunosuppressive principles suggested by single-cell profiling. We propose that these can be organized into a four-layer framework (Figure 1):

Layer 1: Tumor cell-intrinsic neuroendocrine programs. Neuroendocrine differentiation programs—centered on ASCL1-, NEUROD1-, and POU2F3-associated states, with inflamed or non-neuroendocrine programs emerging in specific contexts—may directly reduce immune visibility through downregulation of antigen presentation machinery (HLA-I), suppression of interferon signaling pathways (reported during neuroendocrine transdifferentiation in gastric carcinoma; extended here by analogy) [77], and secretion of immunomodulatory neuropeptides.

Elements of this framework, initially defined in SCLC [63,64], appear to recur in LCNEC [70], cervical NEC [31], ONB [33], prostate NEC [51,99], and PPGL [88], although the degree of conservation likely varies by subtype. Importantly, neuroendocrine-high states often correlate with immune-cold phenotypes, while non-neuroendocrine or inflamed states tend to show greater immune visibility [40,66,70]. These observations suggest that phenotypic plasticity is not merely a tumor-intrinsic adaptation but may also have direct consequences for TME composition and immunotherapy responsiveness. In the evidence-tier terms used in this review, Layer 1 is best regarded as a strongly supported cross-subtype pattern.

Layer 2: Myeloid-dominated immune infiltration with alternative checkpoint expression. Across several NEN subtypes, including pNETs, PitNETs, pulmonary carcinoids, and selected high-grade neuroendocrine carcinomas, macrophages and other myeloid cells frequently represent major immune populations, with functional polarization toward immunosuppressive (M2-like) states [28,35,57,69,81,94]. In well-differentiated GEP-NETs, the specific checkpoint molecules on myeloid cells (VISTA, TIM-3, Galectin-9) differ from the PD-1/PD-L1 axis targeted by current ICIs [101].

This myeloid-centric checkpoint landscape appears recurrent across several NEN subtypes, including well-differentiated GEP-NETs, pituitary NETs, and carcinoids, although broader cross-subtype confirmation remains limited. Layer 2 likewise represents a strongly supported cross-subtype pattern, although therapeutic targeting of its alternative checkpoints remains a hypothesis requiring clinical validation.

Layer 3: CAF-mediated structural and metabolic barriers. CAFs function as physical and functional barriers to immune infiltration in multiple NEN subtypes [45,73,103], with specific subtypes (matrix CAFs, COLEC11+ CAFs, antigen-presenting CAFs) associated with metastasis, potential T-cell trapping, and poor outcomes. The metabolic coupling between CAFs and tumor cells, potentially involving metabolite exchange [68] as well as ApoE-related stromal signaling [56], adds a metabolic dimension to this structural exclusion.

In GI-NETs and SCLC, CAF-mediated barriers may represent an important determinant of immune accessibility alongside tumor-intrinsic immunogenicity. As a category, Layer 3 reflects an established mechanism of immune exclusion in solid tumors that is strongly supported across several NEN subtypes, although the specific NEN CAF subtypes involved still require validation.

Layer 4: Neuroendocrine secretory modulation. The secretory capacity of neuroendocrine cells can directly modulate the TME through tumor-specific mechanisms. Current evidence comes from three principal examples: CGRP-mediated dendritic cell dysfunction in MTC, shown by single-cell profiling and functionally rescued by CGRP receptor antagonism in vitro [94]; macrophage-derived glutamate signaling associated with immunosuppression and tumor cell migration in pNET [35]; and VGF-associated cancer-associated fibroblast activation in SCLC, with combined VGF/MCT1 targeting disrupting tumor–CAF metabolic coupling in preclinical models [68].

Other neuroendocrine secretory products—including chromogranins, somatostatin, and serotonin—have been implicated in immune modulation in non-NEN contexts, but direct single-cell evidence in NENs remains limited. We therefore designate Layer 4 as a *candidate* layer that is distinctive of NEN biology in principle but currently rests on a small number of subtype-specific examples; systematic cross-subtype investigation will be required to establish whether neuroendocrine secretory immunomodulation represents a generally conserved feature. In these terms, Layer 4 currently remains a hypothesis requiring functional or clinical validation.

**Figure 2:**
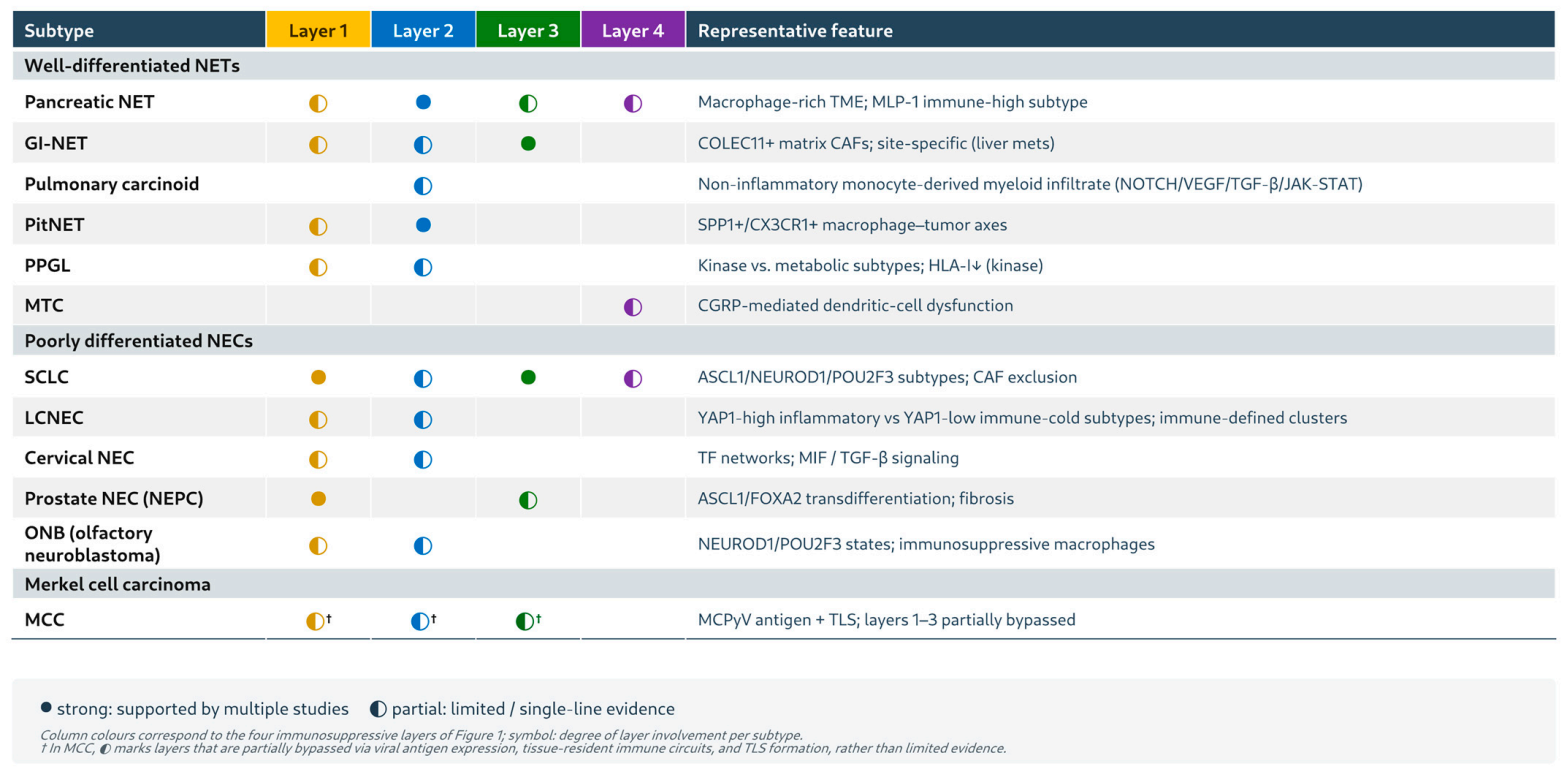
**Involvement of the four immunosuppressive layers across neuroendocrine neoplasm subtypes.** For each subtype, the degree of involvement of each layer (defined in Figure 1) is graded as strong (●; supported by multiple studies) or partial (◐; limited or single-line evidence). Subtypes are grouped into well-differentiated NETs, poorly differentiated NECs, and Merkel cell carcinoma (MCC). Column colours correspond to the four layers of Figure 1, and the Representative feature column summarizes the predominant microenvironmental characteristic of each subtype. Specific supporting studies cited within the figure are indicated for the pulmonary carcinoid myeloid infiltrate [69] and for the YAP1-defined and immune-defined subtypes of LCNEC [70,72]. † In MCC, ◐ denotes layers that are partially bypassed through viral (MCPyV) antigen expression, tissue-resident immune circuits, and tertiary lymphoid structure (TLS) formation, rather than limited evidence.

The relative contribution of each layer likely varies across NEN subtypes. We emphasize that this four-layer framework is intended as a hypothesis-generating conceptual scaffold to guide translational investigation, not an exhaustive or fixed taxonomy of NEN immunosuppression. These subtype-dependent differences, and their contrast between well-differentiated NETs and poorly differentiated NECs, are summarized in Table 2 and Figure 2 and examined in detail in Section 4.6.

### 4.6. Differential Operation of the Four-Layer Framework Across Well-Differentiated NETs and Poorly Differentiated NECs

Building on the layer definitions in Section 4.5, this section contrasts how the four-layer framework operates in well-differentiated NETs versus poorly differentiated NECs, reflecting their distinct underlying biology. The 2022 WHO classification separates well-differentiated NETs (graded G1–G3 by Ki-67 and mitotic count) from poorly differentiated NECs, which include small cell lung cancer (SCLC) and large cell neuroendocrine carcinoma [6]. Notably, G3 NETs and NECs are biologically distinct entities: NECs typically harbor RB1 and TP53/PTEN alterations and follow a more aggressive course, whereas G3 NETs retain the mutational landscape of their lower-grade counterparts [11].

These differences extend to the tumor–immune interface. Well-differentiated NETs are characteristically “immune-cold”, with a low tumor mutational burden (pNETs showing TMB-high rates as low as ~1.3%) [12], limited CD8+ T-cell infiltration [13], generally low and heterogeneous PD-L1 expression [14,15], and largely preserved but immunologically inconspicuous antigen presentation, translating into poor responsiveness to PD-1/PD-L1 blockade (e.g., KEYNOTE-158 objective response rate ~3.7%) [18]. Poorly differentiated NECs, by contrast, tend to carry a higher mutational and neoantigen load, more frequent HLA-I downregulation and interferon-signaling suppression in high-grade contexts [16], and a more variable immune infiltrate, conferring modest but genuine immunogenicity in selected cases.

Layer 1—Tumor cell-intrinsic neuroendocrine programs. In poorly differentiated NEC/SCLC, lineage-defining transcription factors (ASCL1, NEUROD1, POU2F3) and pronounced phenotypic plasticity dominate this layer and are associated with more marked HLA-I downregulation and interferon-signaling suppression [63,64,70]; by extension, a comparable downregulation of interferon pathways has been reported during neuroendocrine transdifferentiation in gastric carcinoma [77]. Neuroendocrine-high, immune-cold states recur in cervical NEC [31], olfactory neuroblastoma [33], and prostate NEC [51,99], whereas non-neuroendocrine or inflamed states show greater immune visibility [40,66,70]. In well-differentiated NETs, by contrast, the dominant programs are organ-appropriate endocrine differentiation states—exemplified by ARX- and PDX1-associated epigenomic subtypes in pNETs [61]—and lineage plasticity appears comparatively limited, so that tumor-intrinsic immune evasion in NETs reflects a stable endocrine identity rather than the de-differentiation and transcriptional rewiring characteristic of NECs.

Layer 2—Myeloid-dominated infiltration with alternative checkpoint expression. In well-differentiated GEP-NETs, the immune infiltrate is frequently myeloid-predominant, and the checkpoint molecules expressed on these cells (VISTA, TIM-3, Galectin-9) differ from the PD-1/PD-L1 axis targeted by current ICIs [101], offering a mechanistic explanation for ICI resistance in this subtype; similar myeloid-centric, M2-polarized patterns are reported across several NETs [28,35,57,69,81,94]. In poorly differentiated NEC/SCLC, the composition and density of the infiltrate are more variable and appear to track with mutational burden and neuroendocrine differentiation state, such that some NECs retain a myeloid-dominant, immunosuppressive milieu while others acquire greater T-cell visibility; broader cross-subtype confirmation in NECs remains limited [28,69].

Layer 3—CAF-mediated structural and metabolic barriers. CAF-driven exclusion has been documented at both ends of the spectrum, including well-differentiated GI-NETs and pNETs as well as SCLC [45,68,73,103]. In NETs, specific CAF subtypes (matrix, COLEC11+, and antigen-presenting CAFs) together with ApoE-related stromal signaling have been linked to T-cell trapping, metastasis, and poor outcome [45,56,73,103], whereas in SCLC the emphasis has been on tumor–CAF metabolic coupling, such as VGF/MCT1-dependent crosstalk [68]. Whether the CAF subtypes, matrix composition, and metabolic-coupling modes differ qualitatively between NETs and NECs—or only in degree—has not yet been systematically compared and remains an open question.

Layer 4—Neuroendocrine secretory modulation. Direct immunomodulation by neuroendocrine secretory products is, to date, supported mainly by examples skewed toward well-differentiated or intermediate NET biology: CGRP-mediated dendritic cell dysfunction in medullary thyroid carcinoma [94] and macrophage-derived glutamate signaling in pNET [35]. A poorly differentiated example also exists, namely VGF-associated CAF activation and metabolic coupling in SCLC [68]. Because the evidence rests on only a few subtype-specific observations, the differential contribution of this layer between NETs and NECs cannot yet be firmly established, and Layer 4 should be regarded as a candidate, NEN-distinctive principle requiring systematic cross-subtype investigation.

Merkel cell carcinoma (MCC) represents an instructive exception to this NET–NEC dichotomy. Although histologically a poorly differentiated cutaneous neuroendocrine carcinoma, virus-positive MCC partially bypasses Layers 1–3 through strong viral (Merkel cell polyomavirus) antigen expression, tissue-resident immune circuits, and tertiary lymphoid structure formation, features that are associated with its comparatively high responsiveness to immune checkpoint inhibitors [44,82,83]. MCC therefore suggests that neuroendocrine lineage does not inevitably dictate immune evasion, and that the relative weight of each layer can be overridden by tumor-specific antigenicity.

Taken together, these comparisons indicate that the relative contribution of each layer is subtype-dependent rather than fixed: tumor-intrinsic plasticity and antigen-presentation defects (Layer 1) together with infiltrate variability (Layer 2) are more prominent in poorly differentiated NECs, whereas multilayered convergence onto a stable immune-cold state is best exemplified by well-differentiated GEP-NETs, and PPGLs display further kinase- versus metabolic-type differences in layer engagement [88]. The four-layer framework is therefore intended as a conceptual scaffold for organizing these subtype-specific patterns and generating testable hypotheses, rather than as a fixed or exhaustive taxonomy.

## 5. Translational Implications for NENs

### 5.1. Prognostic Biomarkers Derived from NEN TME Profiling

Single-cell and spatial transcriptomics have generated a rich pipeline of prognostic and treatment-stratification biomarkers at various stages of development. Table 3 summarizes the current validation status of key biomarkers. Because many of these biomarkers and therapeutic hypotheses are derived from small discovery cohorts, computational inference, or preclinical validation, their clinical readiness varies substantially. Throughout this section, we distinguish established or clinically validated markers from exploratory candidates that require independent validation, prospective testing, or functional confirmation.

At the discovery stage, the pNET MLP-1 subtype [55] may identify an immune-high subset with potential relevance to ICI stratification independent of TMB. However, this finding awaits prospective validation in immunotherapy-treated cohorts. The VISTA/TIM-3 expression pattern on myeloid cells in well-differentiated GEP-NETs [101] represents both a discovery-stage biomarker and a candidate therapeutic target, as discussed in Section 5.2.

At the validation stage, TME-derived immune scoring systems have been tested in independent cohorts. The ISpnet immunoscore for pNETs, incorporating six immune features, predicted relapse-free survival with validation in a separate dataset [111]. Three immune infiltration subtypes in pNETs showed prognostic value with MMP gene involvement in metastatic risk [112].

Pan-cancer TME subtyping has identified four conserved microenvironment subtypes across 20 cancer types, confirmed in 24 The Cancer Genome Atlas (TCGA) tumor entities, and may provide a comparative framework for positioning NENs within broader immunological classifications [137].

For advanced clinical validation, tissue-resident lymphocyte programs in MCC have been associated with immunotherapy efficacy and may be assessable through multiplex immunofluorescence panels [80,138]. TLS quantification by spatial proteomics, particularly in MCC, offers a pathologically assessable biomarker for immunotherapy selection [44,83].

Beyond TME-derived biomarkers, broader molecular stratification factors remain clinically relevant. TMB-high status, while rare in well-differentiated NETs, identifies a subset of patients who benefit from pembrolizumab (ORR 29% vs. 6% in TMB-low) [126], and MSI-H/dMMR status is associated with an ORR of 34.3% across non-colorectal MSI-H/dMMR cancers [127].

### 5.2. Therapeutic Targets in NENs: From Single-Cell Discovery to Clinical Development

#### 5.2.1. DLL3-Directed Therapies

**Figure 3:**
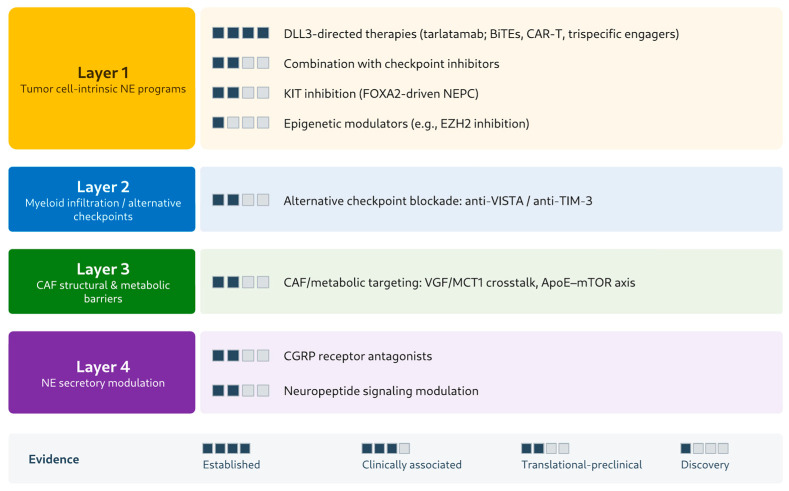
**Layer-matched therapeutic vulnerabilities in neuroendocrine neoplasms.** Therapeutic strategies are organized according to the four-layer framework of Figure 1. Layer 1: DLL3-directed therapies (tarlatamab; BiTEs, CAR-T cells, trispecific engagers) [117,118,119,120,121,122,123,139,140,141,142,143], combination with checkpoint inhibitors [139], KIT inhibition (FOXA2-driven NEPC) [52], and epigenetic modulators (e.g., EZH2 inhibition) [92]. Layer 2: alternative checkpoint blockade (anti-VISTA, anti-TIM-3) [101,113,114,115,116]. Layer 3: CAF and metabolic targeting (VGF/MCT1 crosstalk, ApoE–mTOR axis) [56,68]. Layer 4: CGRP receptor antagonists and neuropeptide signaling modulation [94]. For each strategy, the filled squares indicate the evidence tier—■■■■ Established, ■■■□ Clinically associated, ■■□□ Translational/preclinical, or ■□□□ Discovery—consistent with the grading scheme used in Table 3.

Throughout this section, therapeutic strategies are positioned by evidence level—from established clinical strategies, through clinically associated candidates and translational or preclinical candidates, to discovery-only hypotheses—consistent with the validation status and clinical readiness summarized in Table 3. The corresponding layer-matched strategies and their evidence tiers are summarized in Figure 3.

DLL3 (delta-like ligand 3) represents one of the most advanced therapeutic targets to emerge from the molecular characterization of NENs. DLL3 is a Notch ligand highly expressed on tumor cell surfaces with minimal expression in normal tissues, providing an exceptional therapeutic window [117,118]. Comprehensive analysis has confirmed DLL3 enrichment in pulmonary and prostate neuroendocrine carcinomas, while gastroenteropancreatic NETs show lower expression, necessitating alternative targeting strategies for these subtypes [119].

Tarlatamab, a DLL3/CD3 bispecific T cell engager (BiTE), has progressed through clinical development from promising preclinical data suggesting potent T cell-mediated tumor regression [120] to phase 1 results showing durable responses (ORR 23.4%, median duration of response 12.3 months) in heavily pretreated SCLC [121], to phase 2 results establishing a 40.4% ORR at the 10 mg dose [122], and most recently to phase 3 confirmation of overall survival benefit compared with chemotherapy, with median overall survival of 13.6 versus 8.3 months and a hazard ratio for death of 0.60, alongside fewer grade 3 or higher adverse events (27% vs. 62%) in the tarlatamab group [123].

In SCLC, DLL3-directed bispecific therapy has therefore reached the level of an established clinical strategy—the most clinically mature target to emerge from NEN molecular profiling. Its extension to NEN subtypes beyond SCLC—including extrapulmonary (e.g., gastroenteropancreatic) NEC and prostate NEPC—remains unestablished and, at present, a translational candidate pending subtype-specific validation.

Beyond tarlatamab, the DLL3-targeting pipeline includes IL-18-secreting DLL3-directed CAR-T cells showing enhanced efficacy in combination with anti-PD-1 [139], allogeneic DLL3 CAR-T cells achieving complete responses in SCLC models [140], HPN328 (a trispecific T cell-activating protein) showing dose-dependent cytotoxicity [141], and [89Zr]Zr-DFO-SC16.56 anti-DLL3 positron emission tomography (PET) imaging as a potential companion diagnostic, with DLL3-expressing tumors detectable in 15 of 16 evaluable cases (94%) [142]. BI 764532, another DLL3/CD3 BiTE, is in first-in-human testing for SCLC and NEC [143].

With the exception of tarlatamab, these DLL3-directed agents are not yet established in any NEN setting: the IL-18-secreting and allogeneic CAR-T cells [139,140] and HPN328 [141] remain at the preclinical stage, whereas anti-DLL3 PET imaging [142] and BI 764532 [143] are at an early (first-in-human) clinical stage.

However, DLL3-directed therapies face challenges related to phenotypic plasticity. Simpson et al. reviewed how neuroendocrine-to-non-neuroendocrine switching, including but not limited to NOTCH-related mechanisms, may be associated with reduced DLL3 expression and therapeutic resistance [124]. Chou et al. reported that antigen loss may represent a resistance mechanism in neuroendocrine prostate cancer treated with AMG 757 [125]. These findings help motivate strategies to overcome resistance, including combination with Notch pathway inhibitors or sequential therapeutic approaches.

#### 5.2.2. Alternative Checkpoint Blockade

The predominance of alternative myeloid checkpoints in GEP-NETs has opened new therapeutic avenues. Anti-TIM-3 and anti-VISTA antibodies are now in clinical development for solid tumors, though NEN-specific trials have not yet been initiated. Sabatolimab (anti-TIM-3), in combination with the anti-PD-1 antibody spartalizumab, showed limited but detectable antitumor activity in advanced solid tumors [113], providing a preliminary rationale for further evaluation in neuroendocrine malignancies. The anti-TIM-3 antibody INCAGN02390 demonstrated safety in a phase I solid tumor trial [114], while sabatolimab plus spartalizumab showed limited activity in anti-PD-1-pretreated melanoma and non-small cell lung cancer (NSCLC) patients [115]. KVA12123, a high-affinity anti-VISTA antibody, has shown potent single-agent and combination activity in preclinical models of poorly immunogenic (“immune cold”) tumors [116], and is being evaluated in a phase 1/2 trial in combination with pembrolizumab for advanced solid tumors.

Given that the well-differentiated GEP-NET TME is dominated by VISTA-expressing myeloid cells rather than PD-1-expressing T cells [101], anti-VISTA therapy may represent a biologically plausible therapeutic direction for well-differentiated GEP-NETs that warrants clinical evaluation.

For NENs specifically, alternative checkpoint blockade thus remains an unvalidated translational/preclinical candidate: the available clinical data (sabatolimab [113,115] and INCAGN02390 [114]) derive entirely from non-NEN solid-tumor trials, the KVA12123 evidence is preclinical or in non-NEN solid-tumor testing [116], and no NEN-specific trial has been initiated. The VISTA-dominant myeloid composition of the GEP-NET TME [101] therefore provides a biologically plausible hypothesis for anti-VISTA therapy rather than clinical validation.

CDK4/6 inhibition has emerged as a TME-modulating strategy with potential applicability to NENs. Jerby-Arnon et al. identified a malignant cell program driving T cell exclusion and checkpoint blockade resistance that is suppressed by CDK4/6 inhibition [144]. Ali et al. showed that CDK4/6 inhibition and PD-1 blockade augment nonoverlapping features of T-cell activation, with sequential treatment enhancing antitumor efficacy [145]. In the neuroendocrine context, Wu et al. showed that combined poly(ADP-ribose) polymerase (PARP) and CDK4/6 inhibition suppresses neuroendocrine differentiation markers in prostate cancer models through synergistic p-Rb1-E2F1 axis inhibition [146], suggesting a dual mechanism relevant to NENs: direct anti-tumor activity plus TME immunomodulation.

Notably, trilaciclib (a CDK4/6 inhibitor approved for myelopreservation during SCLC chemotherapy) has been reported to preserve lymphocyte subsets during cytotoxic chemotherapy in preclinical and early clinical studies [147,148], although direct evidence for NK-cell preservation remains limited. Given the prominence of T cell exclusion programs in several NEN subtypes, CDK4/6 inhibitor combinations appear worthy of further clinical investigation.

As a TME-immunomodulatory combination in NENs, however, this rationale rests largely on preclinical and non-NEN studies (T-cell-exclusion reversal and CDK4/6–PD-1 synergy in other tumor types [144,145]; PARP–CDK4/6 effects in prostate cancer models [146]); such CDK4/6-based immunomodulatory combinations therefore remain translational/preclinical candidates not yet tested in NEN-specific trials, distinct from trilaciclib’s established myelopreservation indication.

#### 5.2.3. TME-Modulating Strategies

Single-cell studies have highlighted several candidate TME-modulating strategies with potential relevance to NENs. Anti-SPP1 approaches targeting the SPP1-ITGAV/ITGB1 axis in aggressive PitNETs [28] could disrupt macrophage-mediated invasion. CGRP receptor antagonists may restore dendritic cell function in MTC and other neuroendocrine tumors that secrete CGRP [94], representing a neuroendocrine-specific immunomodulatory strategy that is relatively uncommon in other tumor contexts.

CAF-targeting strategies, including disruption of VGF-associated tumor–CAF crosstalk [68] and the ApoE-mTOR axis [56], may help address stromal barriers to immune infiltration. Preclinical evidence that mTOR inhibition suppresses ApoE-mediated stromal remodeling [56] provides a hypothesis-generating rationale for exploring mTOR inhibitor–immunotherapy combinations in pNETs. KIT inhibition for FOXA2-driven neuroendocrine prostate cancer [52] targets the neuroendocrine transition directly. EZH2 inhibition has been proposed for olfactory neuroblastoma, based on cell-of-origin studies [92], potentially disrupting epigenetic programs that maintain the neuroendocrine state.

These TME-modulating strategies differ in how far they have been experimentally tested: the CGRP–dendritic cell axis has in vitro functional support (receptor antagonism restored DC development [94]); KIT inhibition [52], VGF/MCT1 disruption [68], and ApoE–mTOR targeting [56] rest on preclinical in vivo or model evidence; and the anti-SPP1 [28] and EZH2 [92] approaches remain computationally inferred or discovery-stage proposals. None has yet been tested in an NEN-specific clinical trial, so all remain preclinical or discovery-stage targets awaiting clinical validation.

#### 5.2.4. PPGL-Specific Translational Implications

The molecular subtyping of PPGLs by single-cell approaches has generated subtype-specific translational hypotheses. For kinase-type pheochromocytomas (RET/PNMT-positive), the HLA-I downregulation associated with RET signaling [88] suggests that kinase inhibitors such as cabozantinib may merit evaluation as partners for immunotherapy. Cabozantinib has shown clinical activity across multiple NEN subtypes [105], and combination trials with immunotherapy are ongoing.

For metabolic-type pheochromocytomas (NDUFA4L2/COX4I2-positive), which retain antigen-presenting capacity but harbor immunosuppressive features [88], alternative strategies targeting the metabolic TME or combining anti-angiogenic agents with radionuclide therapy may be more appropriate [106]. Current PPGL management increasingly incorporates tyrosine kinase inhibitors, radionuclide therapy, somatostatin receptor-directed approaches, and selected immunotherapy strategies for metastatic disease [149]. This subtype-guided approach illustrates how single-cell profiling may inform precision-oriented therapeutic hypotheses even in rare endocrine tumors.

In evidence terms, cabozantinib represents a clinically associated candidate (single-agent activity established across NEN subtypes [105], immunotherapy combinations still investigational), whereas the kinase- and metabolic-subtype-guided pairings remain discovery-stage hypotheses.

### 5.3. Treatment Response Prediction in NENs

#### 5.3.1. PRRT Response Prediction

Peptide receptor radionuclide therapy (PRRT) with 177Lu-DOTATATE is a mainstay of treatment for somatostatin receptor-positive NETs, yet patient selection and response prediction remain challenging. TME profiling is emerging as a complementary tool for PRRT optimization.

It is important to distinguish strategies already established in clinical practice—PRRT itself and proliferation- or mutation-based selection (Ki-67 thresholds [131], and the tumor-agnostic MSI-H/dMMR [127] and TMB-high [126] indications)—from the TME- and imaging-derived predictors and PRRT–immunotherapy combinations described below, which remain at the candidate or investigational stage.

Zeng et al. indicated that immune microenvironment parameters—specifically Treg low density and CD8+TIM-3+ low density—predict PRRT response in GEP-NETs [102], providing a biologically grounded patient selection strategy that integrates TME assessment with theranostic decision-making.

Imaging biomarkers, including pre-treatment baseline tumor volume [128], 68Ga-DOTATATE uptake parameters (SUVmean, tumor volume) [129], and machine learning models combining multiple imaging features [130], have been developed for PRRT response prediction and individualized dosimetry. These TME- and imaging-derived predictors are best regarded as translational or clinically associated candidates that require prospective validation before routine use in PRRT selection.

Preclinical models provide a plausible mechanistic basis for PRRT-immunotherapy synergy. Esfahani et al. showed that sequential administration of [177Lu]DOTATATE followed by anti-PD-1 induced the strongest inflammatory response and tumor shrinkage in GEP-NET models, outperforming concurrent administration or reversed sequencing [150]. This finding suggests that PRRT-induced DNA damage triggers immunogenic cell death, creating a window for effective ICI engagement.

Limited clinical support comes from a case-based report by Aicher et al., in which short-interval low-dose PRRT combined with pembrolizumab was associated with renewed antitumor activity in two immunocompromised patients with metastatic MCC who had progressed on prior avelumab [151]. The National Cancer Institute (NCI) consensus conference has formally recommended PRRT-immunotherapy combinations for clinical investigation [152], and the accumulating preclinical and early clinical data support prospective evaluation of this combination in NET-specific clinical trials.

As a combination strategy, PRRT plus immunotherapy is therefore not yet a clinically established option but a future direction requiring prospective evaluation: its rationale rests on preclinical sequencing data [150], a two-patient case-based report in MCC [151], and a consensus recommendation for clinical investigation [152], with no completed NET-specific efficacy trial to date.

Ki-67 stratification remains relevant, with G3 gastroenteropancreatic neuroendocrine neoplasms (GEP-NENs) showing PRRT response rates of 31–41% and disease control rates of 69–78% when Ki-67 is below 55%, with 18F-FDG/SSR dual-tracer imaging aiding patient selection [131]. This proliferation-based selection is already part of routine clinical practice, in contrast to the TME- and imaging-derived predictors above, which remain investigational.

#### 5.3.2. Immunotherapy Response Prediction

Single-cell-derived biomarkers for immunotherapy selection in NENs include the SCLC molecular subtype, in which the inflamed SCLC-I subtype appears to show greater immunotherapy sensitivity [63]; TMB-high status, predicting pembrolizumab response (ORR 29%) [126]; MSI-H/dMMR status, conferring sensitivity across tumor types [127]; TLS density in MCC [44,83]; preexisting tissue-resident T cell circuits in MCC [80]; neuroendocrine differentiation state, with non-neuroendocrine phenotypes showing enhanced immune visibility [40,66]; YAP1 status in LCNEC, stratifying patients for immunotherapy versus SCLC-directed therapy [70]; and the MLP-1 “immune-high” subtype in pNETs, which may represent a candidate biomarker for immunotherapy stratification [55].

Within this list, MSI-H/dMMR status is an established, tumor-agnostic clinical biomarker, and TMB-high is a clinically associated predictor, whereas the single-cell-derived subtype and spatial markers (for example, SCLC-I, YAP1 status, MLP-1, and TLS density) currently remain clinically associated or discovery-stage candidates requiring NEN-specific validation.

The clinical reality of immunotherapy in NENs is one of selective efficacy. Pembrolizumab monotherapy achieves only 3.7% ORR in unselected NETs [18], while dual checkpoint blockade reaches approximately 26% ORR in high-grade neuroendocrine neoplasms [22] and, in a post hoc subgroup analysis of high-grade lesions, 44% ORR [21], with 11% reported in pancreatic NENs [153]. These varying response rates underscore the need for TME-informed patient selection rather than empirical immunotherapy use. Emerging immunotherapy strategies reviewed by Urman et al. include PRRT-ICI combinations, bispecific T cell engagers, CAR-T cells, and tumor vaccines [154], each requiring biomarker-guided selection to optimize the therapeutic ratio in this heterogeneous disease.

### 5.4. Liquid Biopsy and Circulating Biomarkers in NENs

Liquid biopsy platforms represent the most clinically mature translational output of molecular NET characterization. The NETest, a 51-gene blood-based transcript panel, has demonstrated consistently high performance, including diagnostic accuracy of 87% versus 54% for chromogranin A (CgA) and imaging concordance of 91% versus 46% for CgA [132], 97% accuracy for PRRT response prediction through the PRRT Predictive Quotient (PPQ) [134], and high diagnostic accuracy in pulmonary carcinoids [133]. Multiple validation studies across NET subtypes have supported its utility for diagnosis, disease monitoring, and treatment response assessment relative to CgA [133].

The NETseq ensemble classifier, based on peripheral blood RNA-seq, has been reported to achieve 93% sensitivity and 91.4% specificity for detecting PRRT-naïve GEP-NETs and may provide a non-invasive adjunct for early treatment-response monitoring in patients receiving ^177Lu-DOTATATE PRRT, although its role as a definitive binary classifier of PRRT responders and non-responders requires further validation [135].

Circulating tumor cell (CTC) clusters have been identified and characterized in patients with neuroendocrine tumors using nanosubstrate-embedded microchips; preliminary data from the same study showed that dynamic changes in total NET CTC and NET CTC cluster counts during PRRT correlated with clinical responses in a subset of patients, suggesting their potential utility for non-invasive response monitoring, although longitudinal validation is still required [136].

Circulating tumor DNA (ctDNA) methylation profiling, leveraging the pan-tissue DNA methylation atlas by Zhu et al. [54], represents another potential avenue for non-invasive NET subtyping and monitoring, although clinical validation in NET-specific cohorts remains pending.

## 6. Discussion

### 6.1. Integrative Framework: Reframing NEN Immunosuppression Through Single-Cell Technologies

The synthesis of single-cell and spatial transcriptomics data across major NEN subtypes suggests a coherent, multi-layered immunosuppressive architecture that helps move beyond the simplistic “immune cold” designation. The proposed four-layer framework offers a useful conceptual explanation for the heterogeneous immunotherapy responses observed across NEN subtypes. We present this four-layer framework as a hypothesis-generating conceptual scaffold for translational investigation rather than an exhaustive or fixed taxonomy, one that will require systematic cross-NEN validation.

Within this framework, the strength of evidence differs by layer: Layers 1 and 2 (tumor-intrinsic neuroendocrine programs and myeloid-dominated checkpoint biology) are best regarded as strongly supported cross-subtype patterns; Layer 3 (CAF-mediated exclusion) reflects an established mechanism of immune exclusion in solid tumors that is increasingly supported in NENs; and Layer 4 (neuroendocrine secretory immunomodulation) remains a hypothesis requiring functional or clinical validation.

This framework differs from models proposed for other immune-cold tumor types in instructive ways. Glioblastoma, often cited as a paradigm of immune-cold biology, shares a myeloid-dominated TME with extensive microglial and macrophage infiltration and characteristic T-cell exhaustion programs [155,156], but lacks the neuroendocrine-specific Layers 1 and 4 proposed here.

Microsatellite-stable colorectal cancer (MSS-CRC) shares CAF-mediated immune exclusion (Layer 3), exemplified by TGF-β-driven stromal programs that confer resistance to PD-1 blockade [157,158], but typically lacks the alternative myeloid checkpoint dominance observed in well-differentiated GEP-NETs [101]. Pancreatic ductal adenocarcinoma (PDAC) exhibits dense desmoplastic stroma analogous to NEN Layer 3 [159,160], yet its CAF taxonomy—dominated by myofibroblastic and inflammatory CAFs—is molecularly distinct from the matrix CAF and antigen-presenting CAF populations described in NENs [45,73,103].

What may distinguish NENs from other immune-cold tumors is the convergence of all four layers, with Layer 4 (neuroendocrine secretory immunomodulation) representing a relatively distinctive mechanistic feature of this tumor family, although direct mechanistic evidence remains limited to a small number of subtype-specific examples [35,68,94] and warrants systematic cross-NEN validation.

The framework also explains the exceptional responsiveness of MCC to immunotherapy. MCC may partially circumvent Layer 1 through viral antigen expression that enhances tumor immunogenicity, Layer 2 through a relatively balanced myeloid/lymphoid infiltrate including TLS-associated B-cell structures [44,83], and Layer 3 through its skin-based location, which may facilitate tissue-resident T-cell engagement [80]. This “layered escape” model suggests that other NEN subtypes may warrant evaluation of layer-specific therapeutic strategies, such as anti-VISTA approaches for Layer 2, CAF-targeting strategies for Layer 3, or CGRP receptor antagonists for Layer 4.

### 6.2. Clinical Implications: Potential Translation Integration

From a translational perspective, single-cell and spatial transcriptomic findings suggest several clinically relevant themes that may eventually inform biomarker-guided management.

First, the limited activity of PD-1/PD-L1 monotherapy in well-differentiated GEP-NETs may reflect the predominance of alternative myeloid checkpoints not targeted by current ICIs [101]. Although NEN-specific anti-VISTA or anti-TIM-3 clinical trials have not yet been initiated, the preclinical data supporting KVA12123 in immune-cold tumors [116] and one partial response observed in SCLC in the phase I/Ib sabatolimab combination trial [113] provide rationale for further evaluation in neuroendocrine cohorts. These observations motivate a forward-looking trial proposal—the inclusion of neuroendocrine cohorts in future basket trials of novel checkpoint inhibitors—rather than a currently available treatment option, as no NEN-specific anti-VISTA or anti-TIM-3 trial has yet been initiated.

Second, molecular subtyping—particularly the SCLC subtype classification and the REST/YAP1 axis—may help refine therapeutic stratification. SCLC-I and YAP1-high LCNEC appear more likely to show immunotherapy-sensitive features, whereas ASCL1-high tumors may be more relevant to DLL3-directed strategies [63,70]. In pNETs, molecular subtyping may help distinguish tumors with greater immunotherapy relevance from those more closely linked to stromal remodeling pathways that, in preclinical studies, appear to be influenced by mTOR inhibition [55,56]. At present, this molecular subtyping is a research-stage stratification hypothesis rather than a validated clinical tool.

Third, TME-based biomarkers, including immune cell composition, TLS density, and checkpoint expression patterns, may represent useful complementary variables alongside Ki-67, TMB, SSTR status, and molecular subtype in future treatment stratification frameworks. For MCC, spatially defined immune biomarkers such as TLS quantification and tissue-resident immune profiling may help predict immunotherapy outcomes [44,80].

Fourth, circulating molecular biomarkers such as NETest are already clinically available and may complement treatment monitoring and response assessment in well-differentiated NETs [134]. Integration of NETest PPQ scores with TME-derived biomarkers may offer a broader framework for precision-oriented assessment in well-differentiated NETs. This TME-integrated approach, however, remains a prospective concept that has not yet been clinically validated. In PPGLs, molecular classification may help prioritize subtype-matched therapeutic hypotheses [88,105,106].

In evidence terms, therefore, only a minority of these themes—NETest monitoring together with MSI-H/dMMR-, TMB-, Ki-67-, and SSTR-guided management—are part of current clinical practice, while the remainder are prospective directions requiring NEN-specific validation before clinical adoption.

### 6.3. Limitations

#### Several Important Limitations Constrain the Current Evidence Base

Technical and methodological limitations. Single-cell measurements in NENs are shaped by several technical factors that warrant caution. Tissue dissociation and sample handling can alter the recovered cell-type composition and induce stress-response gene-expression artifacts, with dissociation conditions, preservation methods, and processing time each contributing to protocol-specific biases [161,162]; these effects are particularly relevant for the fragile neuroendocrine and stromal populations central to this review. Single-cell and single-nucleus workflows are not directly comparable—snRNA-seq underrepresents cytoplasmic and secretory transcripts while better tolerating frozen or hard-to-dissociate tissue—so the frequent mixing of scRNA-seq and snRNA-seq across NEN studies complicates cross-study comparison of immune and secretory programs.

Batch effects arising from differences in institution, platform chemistry, and processing time add further technical variation that, in the small cohorts typical of NEN studies, is often confounded with biological subtype. Spatial platforms also span a wide resolution range, from multi-cell spots (Visium) to subcellular precision (MERFISH; multiplexed error-robust fluorescence in situ hybridization), which affects the confidence with which cell–cell contacts and niche structures can be assigned. Finally, cell-state annotation remains only partially standardized, as the definitions and thresholds used to label neuroendocrine, myeloid, and CAF states differ between studies, so nominally equivalent cell states may not be strictly comparable across datasets.

Computational inference limitations. Many of the cell–cell communication axes highlighted in this review were derived from computational ligand-receptor analyses (for example, CellChat, NicheNet, and CellPhoneDB). Such methods infer putative interactions from the co-expression of ligand-receptor pairs and, in spatial data, from physical proximity; they therefore support inferred associations rather than establishing functional or causal signaling, which requires experimental perturbation and validation. The same caveat applies to computationally inferred developmental trajectories, regulon activities, and the deconvolution of spot-level spatial data into cell-type proportions.

Accordingly, many of the interaction axes, biomarkers, and therapeutic targets summarized here should be regarded as hypothesis-generating findings that remain at the discovery stage and await independent functional and prospective validation; the experimental strategies needed to close this gap are outlined in Section 7 (Future Directions).

Sampling and cohort limitations. Most NEN single-cell studies involve small cohorts (typically 10–50 patients), which limits statistical power for correlating cellular features with clinical outcomes, and the inherent rarity of many subtypes makes assembling adequately powered, multi-institutional cohorts difficult. Sampling is also uneven across the disease course: primary tumors are profiled far more often than metastases—the setting in which treatment decisions are most critical—and primary-versus-metastatic, inter-subtype, and intratumoral site-to-site heterogeneity further complicate the interpretation of any single dataset.

Most studies are, in addition, cross-sectional, capturing TME snapshots rather than dynamic evolution; evidence that SCLC subtypes undergo temporal evolution under MYC-associated programs [64] and that prostate NE transdifferentiation proceeds through defined stages [51,99] underscores the need for longitudinal, treatment-paired sampling. Finally, the geographic concentration of available studies in Chinese, Western European, and North American institutions may limit generalizability, particularly given documented geographic disparities in NET incidence and survival [4], while the cost and accessibility of these technologies remain barriers to broader and more representative sampling.

Beyond these general constraints, specific subtypes remain underrepresented or absent from the current single-cell literature: to our knowledge, thymic NETs have no published single-cell data, appendiceal NETs lack scRNA-seq profiling, and goblet cell adenocarcinoma (formerly goblet cell carcinoid) of the appendix has not yet been studied at single-cell resolution.

Finally, this is a narrative review with a structured but non-systematic search strategy. Although we aimed to provide a broad and clinically informative synthesis, the rapidly expanding single-cell literature in NENs means that some relevant studies may have been inadvertently omitted. In addition, study selection and thematic synthesis inevitably involved author judgment, and the proposed four-layer framework should therefore be viewed as a conceptual model for future testing rather than a definitive taxonomy of all NEN microenvironments.

### 6.4. Comparison with Other Tumor Types

The NEN TME shares some features with other “cold” tumors, including glioblastoma and microsatellite-stable colorectal cancer, particularly in the dominance of myeloid-mediated immunosuppression.

However, NENs possess unique features—most notably a candidate direct immunomodulatory capacity of neuroendocrine secretory programs, including neuropeptide-related signaling [35,68,94], which currently rests on only a few subtype-specific examples (CGRP in MTC, glutamate in pNET, and VGF in SCLC) rather than an established cross-NEN feature, and the extreme phenotypic plasticity governed by neuroendocrine transcription factor programs [64].

The conserved pan-cancer TME subtypes identified by Bagaev et al. across 20 cancer types, with confirmation in TCGA tumor entities [137], provide a framework for positioning specific NEN subtypes along an immune continuum, from the favorable (immune-rich MCC) to the unfavorable (immune-desert well-differentiated GEP-NETs).

The comparison with neuroendocrine transdifferentiation in non-NET cancers is particularly instructive. In prostate cancer, NE transdifferentiation following androgen deprivation therapy involves transcriptional programs related to those seen in primary NENs [51,98,99], and in gastric cancer, NE differentiation is accompanied by interferon pathway downregulation [77]. These parallels suggest that some immunosuppressive mechanisms identified in primary NENs may also be relevant to acquired NE phenotypes in other cancer types, thereby extending the conceptual relevance of NEN single-cell studies beyond neuroendocrine oncology.

The identification of CRATER niches—tumor cell surface niches associated with antigen presentation and T-cell engagement that predict immunotherapy response in melanoma and may be conceptually relevant to other solid tumors [107]—suggests that spatial analysis of analogous structures in NENs may yield additional predictive biomarkers. Whether the characteristically dense vasculature of many well-differentiated NETs facilitates or impedes the formation of such immune-permissive niches remains to be determined.

## 7. Future Directions for NEN Research

Based on the synthesis above, several major gaps emerge in the current NEN single-cell and spatial transcriptomics literature. These include the lack of harmonized cross-subtype atlases, limited longitudinal and treatment-paired datasets, underrepresentation of rare and metastatic NEN subtypes, insufficient functional validation of computationally inferred cell–cell interactions, and the absence of clinically standardized spatial or transcriptomic biomarkers. Addressing these gaps will require coordinated multi-institutional efforts that combine multi-modal profiling, prospective sampling, functional validation, and biomarker-driven clinical trial design.

Multi-modal integration and data sharing. The combination of scRNA-seq, spatial transcriptomics, ATAC-seq, proteomics, and metabolomics within single studies is likely to provide one of the most comprehensive views of NEN biology. The integration of epigenomic profiling, including enhancer-based approaches such as H3K27ac ChIP-seq [61] with spatial transcriptomics [28] represents the current frontier, but the incorporation of spatial proteomics [44] and spatial metabolomics will further refine our understanding of the NEN TME. Advanced computational frameworks such as SIMVI [163], which disentangles intrinsic and spatially induced cellular states, and NEPAL [164], a neuroendocrine prostate cancer (NEPC)-focused computational tool for identifying neuroendocrine markers from transcriptomic data, will be essential for interpreting these multi-dimensional datasets and, where applicable, for extending such approaches to other NEN subtypes.

Equally important is the deposition of NEN single-cell data into public repositories (such as TISCH2, Single Cell Portal, and the Human Cell Atlas) to enable cross-study meta-analyses and facilitate discovery in this rare disease space.

Longitudinal single-cell analysis. Nearly all current NEN single-cell studies are cross-sectional. Longitudinal profiling of the TME through the course of disease—from primary tumor to metastasis, from treatment-naive to post-therapy—is critically needed. Evidence that neuroendocrine transdifferentiation can proceed through defined temporal stages in prostate cancer [51,99] highlights the potential value of time-resolved analysis. Pre- and post-treatment biopsies analyzed by scRNA-seq and spatial transcriptomics could reveal mechanisms of acquired resistance and identify optimal treatment sequencing strategies.

TME dynamics around therapeutic interventions. Understanding how existing NEN therapies (somatostatin analogues, mTOR inhibitors, anti-angiogenic agents, PRRT) remodel the TME may help inform rational combination. Preclinical evidence that mTOR inhibition suppresses ApoE-mediated stromal remodeling [56] illustrates how mechanistic TME studies may help generate hypotheses for synergistic combinations. PRRT-induced immunogenic effects on the tumor microenvironment deserve systematic single-cell characterization, building on preclinical evidence suggesting that sequential PRRT followed by ICI may induce the strongest inflammatory response [150]. PRRT-ICI combination studies should incorporate paired TME profiling to identify biomarkers of synergy [152].

Artificial intelligence and machine learning. AI-driven integration of multi-modal data—combining scRNA-seq, spatial transcriptomics, radiomics, and clinical data—holds promise for developing comprehensive predictive models. Machine learning dosimetry for PRRT [130] represents an early application, but more sophisticated approaches could integrate TME features with imaging and circulating biomarkers for truly personalized treatment planning. In the future, foundation models trained on large-scale single-cell atlases may help infer aspects of TME composition from more accessible data modalities, potentially lowering the cost barrier to TME-informed clinical decision-making.

Clinical trial design integration. Future NEN clinical trials would benefit from incorporating single-cell and spatial transcriptomics into correlative science programs. Specific priorities include the prospective validation of alternative checkpoint expression, incorporation of molecular subtyping into trial stratification, paired spatial profiling to assess pharmacodynamic effects, and development of serially monitorable liquid-biopsy surrogates. The emerging subcellular-resolution spatial technologies (MERFISH, CosMx) have not yet been widely applied to NENs, but when introduced into this field, they could further refine our understanding of cell–cell interactions in the NEN TME.

Functional validation of inferred cell–cell interactions. As emphasized among the limitations of the current evidence base (Section 4.4 and Section 6.3), many candidate ligand-receptor axes identified by single-cell and spatial analyses—including the macrophage-tumor (for example, SPP1-ITGAV/ITGB1 and INHBA-ACVR1B), CAF-tumor, endothelial-stromal, and neuropeptide-immune interactions summarized in this review—remain computationally inferred. Future studies should prioritize functional validation using organoid co-culture systems, patient-derived xenografts, genetically engineered mouse models, spatial perturbation approaches, and ex vivo tumor slice cultures. Such validation will be essential to distinguish correlative spatial proximity from causal mechanisms and to prioritize therapeutically actionable interactions.

## 8. Conclusions

Single-cell RNA sequencing and spatial transcriptomics have substantially advanced our understanding of the neuroendocrine neoplasm microenvironment. The long-standing characterization of NENs, particularly well-differentiated NETs, as uniformly “immune cold” is increasingly being replaced by a more nuanced, multi-layered view of immunosuppression that appears to vary by organ, histological grade, and molecular subtype. Key insights include myeloid-dominant immunosuppression, CAF-mediated immune exclusion, phenotype-dependent immune visibility, and the candidate mechanism of direct immune modulation by neuroendocrine secretory products, which currently rests on only a few subtype-specific examples (MTC, pNET, and SCLC). These insights are increasingly being aligned with translational and clinical advances, most notably the development of DLL3-directed bispecific antibodies with phase 3 overall survival benefit in SCLC, as well as continued clinical development of alternative checkpoint-targeting strategies in solid tumors with potential relevance to neuroendocrine neoplasms. The proposed four-layer framework may provide a useful, hypothesis-generating conceptual scaffold for translational investigation across NEN subtypes, rather than an exhaustive or fixed taxonomy. Future progress will depend on harmonized multi-institutional atlases, longitudinal treatment-paired sampling, functional validation of inferred interaction networks, and the prospective incorporation of spatial and transcriptomic biomarkers into NEN clinical trials. Broader integration of multi-modal single-cell technologies into clinical trial design, longitudinal monitoring, and data-driven clinical research may help realize the translational potential of these discoveries and ultimately contribute to improved outcomes for patients with neuroendocrine neoplasms.

## Figures and Tables

**Table 1:** **Single-cell and spatial transcriptomic studies in neuroendocrine neoplasms, by subtype.** For each NEN subtype, the predominant single-cell/spatial technologies, sample types, approximate number of studies discussed in this review, representative findings relevant to the cross-organ immunosuppressive principles synthesized in Section 4, and references are listed. Study counts are approximate.

NEN Subtype	Single-Cell/Spatial Technology	Sample Type	Studies (n)	Representative Findings	Ref.
Pancreatic NET	scRNA-seq, snRNA-seq, spatial (Visium HD), proteomics, epigenomics	Primary + metastatic	~10	MLP-1 immune-high subtype; macrophage-derived glutamate; ApoE–CAF stromal remodeling; ARX/PDX1 epigenomic subtypes	[16,24,25,35,39,50,55,56,57,58,59,60,61]
SCLC	scRNA-seq, spatial proteo-transcriptomics	Primary + metastatic	~10	ASCL1/NEUROD1/POU2F3 (+ inflamed SCLC-I) subtypes; CAF-driven immune exclusion; REST/non-NE immune-active states	[26,27,40,45,62,63,64,65,66,67,68]
Pulmonary carcinoids and LCNEC	scRNA-seq, spatial transcriptomics	Primary	4	Non-inflammatory myeloid infiltrate (carcinoids); YAP1 stratifies LCNEC immunogenicity	[69,70,71,72]
Gastrointestinal NETs/NECs (small-intestinal, gastric, colorectal)	scRNA-seq, immune profiling	Primary + metastatic (liver)	~9	COLEC11+ matrix CAFs in liver metastases; site-specific immunity; gastric NE-transdifferentiation interferon downregulation	[15,41,73,74,75,76,77,78,79]
Merkel cell carcinoma	scRNA-seq, spatial transcriptomics, spatial proteomics, multimodal	Primary ± metastatic	~6	Tissue-resident memory and Vδ1 γδ T cells predict ICI response; TLS; MCPyV small T–IRF9 interferon suppression	[42,44,80,81,82,83]
Pituitary NET	scRNA-seq, spatial transcriptomics	Primary	~6	SPP1+ and CX3CR1+ macrophage–tumor axes; TIME subtypes; recurrence-state markers	[28,29,38,84,85,86]
Pheochromocytoma/paraganglioma	scRNA-seq, snRNA-seq	Primary + metastatic	~7	Kinase (HLA-I-deficient) vs. metabolic subtypes; broadly immunosuppressive microenvironment	[34,36,87,88,89,90,91]
Olfactory neuroblastoma	scRNA-seq	Primary	4	SCLC-like NEUROD1/POU2F3 states; immunosuppressive macrophages	[32,33,92,93]
Medullary thyroid carcinoma	scRNA-seq (+ in vitro functional)	Primary	1	CGRP-mediated dendritic-cell dysfunction (rescued in vitro)	[94]
Cervical NEC	scRNA-seq, organoid	Primary	~5	Tissue-specific transcription-factor networks; MIF/TGF-β immunosuppression; HPV/SOX2 origin	[30,31,95,96,97]
Prostate NEC (NEPC)	scRNA-seq, multi-omics, Visium spatial	Primary/models	~5	ASCL1/FOXA2-driven transdifferentiation; fibrosis and vascular remodeling in NE regions	[37,51,52,98,99]
Neuroendocrine bladder cancer	Multimodal (scRNA-seq)	Primary	1	Immune-excluded/desert phenotype; immune-infiltrated mixed-histology subset	[100]
MiNEN	Spatial transcriptomics	Primary	1	Morphological compartments align with transcriptomic profiles	[43]

**Table 2:** **Comparison of the four-layer immunosuppression framework between well-differentiated NETs and poorly differentiated NECs.** The table contrasts baseline tumor–immune features and the relative contribution of each of the four layers (Figure 1 and Figure 2) across well-differentiated NET, poorly differentiated NEC (including SCLC), and Merkel cell carcinoma (MCC) as a reference cutaneous NEC. For each feature and layer, the cell summarizes the predominant mechanism, representative subtype(s), and supporting reference numbers used elsewhere in this review, highlighting how each layer differs in action between NET and NEC.

Feature/Layer	Well-Differentiated NET	Poorly Differentiated NEC (Incl. SCLC)	MCC (Reference)
Differentiation/grade	Well-differentiated; low Ki-67 (G1–G2); GEP-NET, pNET, pulmonary carcinoid	Poorly differentiated, high-grade; SCLC, LCNEC, cervical/prostate NEC	Cutaneous poorly differentiated NEC; frequently MCPyV-positive
TMB	Characteristically low; pNET TMB-high rate as low as ~1.3% [12]	Higher (e.g., smoking-related in SCLC); greater neoantigen load	Bimodal: UV-driven high (virus-negative) vs. viral-antigen–driven (MCPyV+) [82]
HLA-I/antigen presentation	Generally retained but low immune visibility; PD-L1 low/heterogeneous [14,15]	HLA-I downregulation, especially in higher-grade/poorly differentiated contexts [16]; IFN signaling suppression [77]	Type I IFN suppression via MCPyV small T–IRF9 axis in virus-positive tumors [82]
Immune infiltration	Immune-cold; limited CD8+ infiltration [13]; myeloid-dominant	Variable; NE-high (ASCL1/NEUROD1/POU2F3) states immune-cold, non-NE/inflamed states more visible [40,66,70]	Comparatively immune-hot; TLS and organized B/T neighborhoods linked to ICI response [44,83]
ICI sensitivity	Low; KEYNOTE-158 ORR as low as ~3.7% [18]	Modest; benefit largely confined to subsets	High responsiveness to immune checkpoint inhibitors [44,83]
Layer 1—Tumor cell-intrinsic NE programs	NE-high immune-cold programs converge to a markedly immune-cold TME (clearest in GEP-NET) [40,66,70]	Lineage TFs defined in SCLC [63,64]; recur in LCNEC [70], cervical NEC [31], ONB [33], prostate NEC [51,99]; HLA-I/IFN suppression [77]	Partially bypassed through viral antigen expression [82]
Layer 2—Myeloid-dominated infiltration/alternative checkpoints	TAM/MDSC M2-like polarization; alternative checkpoints VISTA/TIM-3/Galectin-9 dominate over PD-1/PD-L1 in GEP-NET [101]; myeloid-dominant [28,35,57,69,81,94]	Myeloid infiltration in high-grade NEC; broader cross-subtype confirmation limited [28,69]	Immunosuppressive TAM subsets (CD163+/CD14+/S100A8+) enriched even with substantial CD8+ infiltration [81]
Layer 3—CAF structural and metabolic barriers	CAF physical/metabolic barriers in GI-NET; matrix/COLEC11+/antigen-presenting CAFs, T-cell trapping [45,73,103]; ApoE stromal signaling [56]	CAF-mediated barriers in SCLC; VGF/MCT1 metabolic coupling between tumor and CAF [68]	Less prominent; immune access less CAF-restricted (Layers 1–3 partially bypassed)
Layer 4—Neuroendocrine secretory modulation	Macrophage-derived glutamate signaling in pNET [35]; CGRP-mediated DC dysfunction in MTC [94]	VGF-associated CAF activation/metabolic coupling in SCLC [68]	Limited direct single-cell evidence; candidate principle

**Table 3:** **Candidate biomarkers and therapeutic targets derived from NEN tumor microenvironment profiling.** The Evidence tier column grades each entry as (1) Established clinical biomarker/strategy, (2) Clinically associated candidate, (3) Translational/preclinical candidate, or (4) Discovery-only hypothesis, assigned from the validation status and clinical readiness shown.

Biomarker/ Target	Main NEN Subtype(s)	Evidence Source	Evidence Tier	Validation Status	Clinical Readiness	Key Limitation	Ref.
MLP-1 immune-high subtype	Pancreatic NET	Transcriptomic and immune-signature analysis of pNET cohorts	Discovery-only	Discovery/retrospective validation	Exploratory biomarker for ICI stratification	Requires prospective validation in immunotherapy-treated pNET cohorts	[55]
132-gene immune signature	Pancreatic NET	Molecular subtype analysis with immune profiling	Discovery-only	Discovery	Exploratory prognostic/treatment-stratification marker	Clinical assay and treatment-predictive utility remain unestablished	[55]
ISpnet immunoscore	Pancreatic NET	Immune-feature-based prognostic model	Clinically associated	Validation in independent dataset	Promising prognostic biomarker	Requires broader external validation and clinical implementation studies	[111]
Immune infiltration subtypes with MMP gene involvement	Pancreatic NET	Transcriptomic immune-subtyping studies	Clinically associated	Validation-stage evidence	Prognostic/metastasis-risk stratification candidate	Requires standardization and prospective validation	[112]
VISTA/TIM-3/Galectin-9 myeloid checkpoint pattern	Well-differentiated GEP-NET	scRNA-seq of GEP-NET immune microenvironment	Translational/preclinical	Discovery (NEN); early-phase non-NEN clinical and preclinical therapeutic data	Candidate therapeutic target and biomarker	No NEN-specific therapeutic trial; targetability in NEN remains unproven	[101,113,114,115,116]
Myeloid-dominant immunosuppression	pNET, PitNET, pulmonary carcinoid, MCC, selected NECs	scRNA-seq and spatial immune profiling	Discovery-only	Recurrent discovery across subtypes	Conceptual biomarker of immune-cold/immune-excluded TME	Cell-state definitions and clinical thresholds are not standardized	[28,35,57,69,81,94]
SPP1+ tumor-associated macrophage axis	PitNET; potentially broader NEN relevance	scRNA-seq/cell–cell interaction analysis	Discovery-only	Discovery with mechanistic implication	Candidate macrophage-tumor interaction target	Requires functional and therapeutic validation in NEN models	[28]
CX3CR1+ macrophage/INHBA–ACVR1B axis	PitNET	scRNA-seq ligand-receptor analysis	Discovery-only	Discovery	Candidate microenvironmental interaction marker	Clinical relevance and targetability remain unclear	[29]
CGRP-mediated dendritic cell dysfunction	Medullary thyroid carcinoma	scRNA-seq and in vitro functional restoration with CGRP receptor antagonism	Translational/preclinical	Discovery with functional validation	Candidate neuroendocrine-specific immunomodulatory target	Requires in vivo and clinical validation	[94]
CAF-rich immune-exclusion pattern	SCLC, GI-NET, pNET, NEPC	Single-cell and spatial transcriptomic studies	Discovery-only	Recurrent discovery across subtypes	Candidate marker of immune exclusion and stromal-targeting vulnerability	CAF subtypes and scoring systems require standardization	[37,45,56,67,68,73]
COLEC11+ matrix CAFs	Colorectal NET liver metastasis	scRNA-seq of metastatic colorectal NET	Discovery-only	Discovery	Candidate prognostic and stromal-targeting marker	Needs validation in larger and independent metastatic NET cohorts	[73]
ApoE–tip endothelial cell–CAF axis	Pancreatic NET	Preclinical and microenvironmental studies	Translational/preclinical	Preclinical/mechanistic	Candidate stromal-remodeling pathway	Clinical relevance and druggability require validation	[56]
Tissue-resident CD8+ T cells	Merkel cell carcinoma	Multimodal immune profiling of MCC samples	Clinically associated	Clinical association	Promising ICI response biomarker	Requires standardized assay and prospective validation	[80]
Vδ1 γδ T cells	Merkel cell carcinoma	Multimodal immune profiling	Clinically associated	Clinical association	Candidate ICI response biomarker	Functional contribution and assay standardization remain unresolved	[80]
Tertiary lymphoid structures	Merkel cell carcinoma; potentially other NENs	Spatial proteomic and immune-neighborhood studies	Clinically associated	Clinical association/advanced translational evidence in MCC	Promising spatial biomarker for ICI response	Standardized TLS scoring and cross-NEN validation are needed	[44,83]
SCLC-I/inflamed SCLC subtype	SCLC	Transcriptomic molecular subtyping and clinical correlation	Clinically associated	Clinically relevant subtype framework	Candidate predictor of ICI sensitivity	Prospective biomarker-guided treatment evidence remains limited	[63]
REST-high/reduced-neuroendocrine immune-active state	SCLC	Single-cell and spatial proteo-transcriptomic profiling	Translational/preclinical	Discovery/clinical association	Candidate marker of enhanced antitumor immunity	Requires prospective validation and assay simplification	[40]
YAP1-high inflammatory/mesenchymal phenotype	LCNEC, SCLC-related high-grade NENs	Transcriptomic and immune-subtyping studies	Translational/preclinical	Discovery/translational association	Candidate marker for immunotherapy-sensitive phenotype	YAP1-based classification remains context-dependent	[64,70]
Neuroendocrine differentiation state	SCLC, LCNEC, NEPC, cervical NEC, ONB	Single-cell/multi-omics lineage-state studies	Discovery-only	Recurrent discovery	Candidate stratifier for immune visibility and lineage-directed therapy	Dynamic plasticity complicates static biomarker use	[31,33,40,51,63,64,66,70,77,98,99]
DLL3 expression	SCLC, pulmonary NEC, prostate NEPC, selected NECs	Molecular profiling and clinical DLL3-targeted therapy trials	Established (SCLC); Translational (other NECs)	Clinically actionable in SCLC	High clinical readiness in SCLC; candidate in other NECs	Expression heterogeneity and antigen loss may drive resistance	[117,118,119,120,121,122,123,124,125]
Tarlatamab response	SCLC	Phase 1–3 clinical trials of DLL3/CD3 bispecific antibody	Established	Clinically validated in SCLC	Established therapeutic strategy in SCLC context	Biomarker refinement and applicability to extrapulmonary NECs remain under study	[120,121,122,123]
TMB-high status	NENs, rare in well-differentiated NETs	Tumor-agnostic pembrolizumab evidence and NEN subgroup data	Established	Clinically established tumor-agnostic biomarker	Clinically actionable when present	Low prevalence in well-differentiated NETs limits utility	[12,126]
MSI-H/dMMR status	NENs, selected high-grade or rare cases	Tumor-agnostic immunotherapy evidence	Established	Clinically established tumor-agnostic biomarker	Clinically actionable when present	Rare in most NEN subtypes	[127]
Treg-low and CD8+TIM-3+ low density	GEP-NET treated with PRRT	Immune microenvironment analysis associated with PRRT response	Clinically associated	Clinical association	Candidate PRRT response biomarker	Needs prospective validation and standardized cutoffs	[102]
SSTR imaging parameters	SSTR-positive NETs	Imaging biomarker studies using ^68Ga-DOTATATE and tumor-volume metrics	Established	Clinical/translational validation	Useful adjunct for PRRT selection and response prediction	Imaging thresholds and integration with TME biomarkers remain variable	[128,129]
Machine-learning imaging models for PRRT response	SSTR-positive NETs	Imaging-feature-based predictive modeling	Translational/preclinical	Translational validation	Candidate adjunct for individualized PRRT prediction	Requires external validation and integration with molecular biomarkers	[130]
Ki-67 < 55% with dual-tracer imaging context	G3 GEP-NEN	Clinical PRRT outcome studies	Established	Clinical evidence	Useful for PRRT candidate selection	Does not fully capture TME or molecular heterogeneity	[131]
NETest	Well-differentiated NETs, pulmonary carcinoids, GEP-NETs	Blood-based 51-gene transcript panel validation studies	Established	Advanced clinical validation	Most mature liquid biopsy platform among NET biomarkers	Implementation, external reproducibility, and clinical workflow integration remain issues	[132,133]
PRRT Predictive Quotient	SSTR-positive NETs receiving PRRT	NETest-derived response prediction studies	Clinically associated	Advanced translational/clinical validation	Candidate PRRT response prediction tool	Requires broader prospective confirmation across treatment settings	[134]
NETseq	PRRT-naïve GEP-NET	Peripheral blood RNA-seq classifier	Translational/preclinical	Exploratory/early validation	Non-invasive adjunct candidate	Not yet established as definitive responder/non-responder classifier	[135]
Circulating NET CTC clusters	NETs undergoing PRRT	Liquid biopsy/microchip-based CTC studies	Discovery-only	Exploratory	Candidate non-invasive response-monitoring biomarker	Small cohorts and longitudinal validation needed	[136]
ctDNA methylation profiling	NETs/NENs broadly	Pan-tissue methylation atlas–based concept	Discovery-only	Exploratory	Candidate non-invasive subtyping/monitoring tool	NET-specific clinical validation remains pending	[54]
PPGL metabolic subtype	PPGL	Molecular and immune subtype analysis	Translational/preclinical	Discovery/translational association	Candidate subtype for non-ICI or metabolic/anti-angiogenic strategies	Requires prospective therapy-linked validation	[88,89]
PPGL kinase subtype with HLA-I downregulation	PPGL	Molecular subtype and immune profiling	Translational/preclinical	Discovery/translational association	Candidate for kinase inhibitor–immunotherapy hypotheses	Therapeutic combination remains unproven in prospective trials	[88]

## Data Availability

Data sharing is not applicable to this article because no new datasets were generated or analyzed in this narrative review.

## Abbreviations

ACTH, adrenocorticotropic hormone; ADC, antibody-drug conjugate; ApoE, apolipoprotein E; ASCL1, achaete-scute homolog 1; ATAC-seq, assay for transposase-accessible chromatin with sequencing; BiTE, bispecific T-cell engager; CAF, cancer-associated fibroblast; CAR-T, chimeric antigen receptor T cell; CCR2, C-C chemokine receptor type 2; CGRP, calcitonin gene-related peptide; CRH, corticotropin-releasing hormone; CSF1R, colony-stimulating factor 1 receptor; CTC, circulating tumor cell; ctDNA, circulating tumor DNA; DC, dendritic cell; DLL3, delta-like ligand 3; ECM, extracellular matrix; EZH2, enhancer of zeste homolog 2; FAP, fibroblast activation protein; FOXA2, forkhead box A2; GEP-NEN, gastroenteropancreatic neuroendocrine neoplasm; GEP-NET, gastroenteropancreatic neuroendocrine tumor; GI-NET, gastrointestinal neuroendocrine tumor; HEV, high endothelial venule; HLA-I, human leukocyte antigen class I; HPV, human papillomavirus; ICI, immune checkpoint inhibitor; IDO1, indoleamine 2,3-dioxygenase 1; IHC, immunohistochemistry; INHBA, inhibin subunit beta A; KIT, KIT proto-oncogene receptor tyrosine kinase; LCNEC, large cell neuroendocrine carcinoma; LDHA, lactate dehydrogenase A; MANEC, mixed adenoneuroendocrine carcinoma; MCC, Merkel cell carcinoma; MCPyV, Merkel cell polyomavirus; MCT1, monocarboxylate transporter 1; MDSC, myeloid-derived suppressor cell; MERFISH, multiplexed error-robust fluorescence in situ hybridization; MiNEN, mixed neuroendocrine–non-neuroendocrine neoplasm; MLP-1, metastasis-like primary 1; MSI-H/dMMR, microsatellite instability-high/mismatch repair-deficient; MSS-CRC, microsatellite-stable colorectal cancer; MTC, medullary thyroid carcinoma; mTOR, mechanistic target of rapamycin; NCI, National Cancer Institute; NE, neuroendocrine; NEC, neuroendocrine carcinoma; NEN, neuroendocrine neoplasm; NEPC, neuroendocrine prostate cancer; NET, neuroendocrine tumor; NEUROD1, neuronal differentiation 1; NSCLC, non-small cell lung cancer; ONB, olfactory neuroblastoma; ORR, objective response rate; PARP, poly(ADP-ribose) polymerase; PDAC, pancreatic ductal adenocarcinoma; PDGFR, platelet-derived growth factor receptor; PET, positron emission tomography; PitNET, pituitary neuroendocrine tumor; pNET, pancreatic neuroendocrine tumor; POU2F3, POU class 2 homeobox 3; PPGL, pheochromocytoma and paraganglioma; PRRT, peptide receptor radionuclide therapy; SCLC, small cell lung cancer; SCLC-A/N/P/I, ASCL1/NEUROD1/POU2F3/inflamed subtypes of SCLC; scRNA-seq, single-cell RNA sequencing; SEER, Surveillance, Epidemiology, and End Results; SI-NET, small intestinal neuroendocrine tumor; SIGLEC10, sialic acid-binding immunoglobulin-like lectin 10; snRNA-seq, single-nucleus RNA sequencing; SPP1, secreted phosphoprotein 1; SSTR, somatostatin receptor; TAM, tumor-associated macrophage; TCGA, The Cancer Genome Atlas; TIM-3, T-cell immunoglobulin and mucin-domain containing-3; TIME, tumor immune microenvironment; TKI, tyrosine kinase inhibitor; TLS, tertiary lymphoid structure; TMB, tumor mutational burden; TME, tumor microenvironment; Treg, regulatory T cell; VGF, neurosecretory protein VGF; VISTA, V-domain Ig suppressor of T-cell activation; WHO, World Health Organization; γδ T cell, gamma-delta T cell.
